# Impacts of no-tillage management on nitrate loss from corn, soybean and wheat cultivation: A meta-analysis

**DOI:** 10.1038/s41598-017-12383-7

**Published:** 2017-09-21

**Authors:** Stefani Daryanto, Lixin Wang, Pierre-André Jacinthe

**Affiliations:** Department of Earth Sciences, 723 West Michigan St, SL118, Indiana University Purdue University Indianapolis, Indianapolis, IN 46202 USA

## Abstract

Although no-till (NT) has been promoted as an alternative land management practice to conventional tillage (CT), its impact on water quality, especially nitrate (NO_3_^−^) loss remain controversial. We conducted a meta-analysis to compare NO_3_^−^ concentration and load in NT and CT systems via two major transport pathways: runoff and leaching. Rainfall variability, aridity, soil texture, tillage duration, crop species, and fertilizer type were used as co-varying factors. In comparison to CT, NT resulted in an overall increase of runoff NO_3_^−^ concentration, but similar runoff NO_3_^−^ load. In contrast, leachate NO_3_^−^ load was greater under NT than under CT, although leachate NO_3_^−^ concentration was similar under both tillage practices, indicating that the effect of NT on NO_3_^−^ load was largely determined by changes in water flux. Some deviations from these overall trends, however, were recorded with different co-varying variables. In comparison to CT, NT, for example, generated lower leachate NO_3_^−^ concentration and similar (instead of elevated) NO_3_^−^ leachate load from soybean fields (no N fertilizer applied). These results suggest NT needs to be complemented with other practices (e.g., cover crops, reduced N rate, split N application) in order to improve soil N retention and water quality benefits.

## Introduction

Nitrate (NO_3_^−^) is the primary form of nitrogen (N) loss from agricultural settings and has been an important contributor to hypertrophic or eutrophic conditions^1–4^. Due to its mobility, water solubility, and persistency, particularly in the presence of oxygen, NO_3_^−^ has long been recognized as a widespread water pollutant. The World Health Organization (WHO) recommends an MCL (maximum concentration limit) of 50 mg NO_3_^−^ L^−1^ in public water supplies. In addition, under oxygen-limited conditions, NO_3_^−^ readily undergoes denitrification, resulting in the emission of nitrous oxide – a greenhouse gas^3^.

During the last few decades, agricultural practices that aim to mitigate N loss from croplands have been evaluated, including the retention of crop residue on the soil surface, the use of cover crops during fallow period, and better synchronization between fertilizer application and crop N demand^5^. Collectively, these practices are referred to as ‘conservation agriculture’, with no-tillage (NT or zero tillage) as the foundational basis for improved management of N cycling in agro-ecosystems^5^. In contrast to conventional land management (i.e., conventional tillage or CT), NT is an agricultural practice that leaves crop residue on the soil surface and limits soil disturbance (except for small slits to add fertilizer)^6^. The use of NT practice has gained popularity in US, South America and other world regions. In 2000/2001, about 21% (~13.5 × 10^6^ ha), 32% (~9.25 × 10^6^ ha) and 52% (~0.96 × 10^6^ ha) of total croplands in Brazil, Argentina and Paraguay were under NT management. In the US, an estimated 20% of all croplands (~22.3 × 10^6^ ha) has been under NT management^7^ with an estimated area increase of 1.5% per year^6^.

In general, NT management offers several advantages when compared to CT as it improves various aspects of the crop-soil relationships (e.g., accumulation of organic matter, improved water retention and infiltration, moderation of soil temperature). NT practices can significantly reduce soil erosion and runoff but, at the same time, can increase water infiltration^8^. With the amelioration in soil organic matter (SOM) content, vegetative growth and fertilizer-use efficiency are generally better for crops grown under NT management compared to CT^9^. Since the load of agricultural nutrients transported to surface- and groundwater is a function of water volume and pollutant concentration (load = concentration × water volume)^10^, NT practice is therefore expected to affect nutrient export due to its effect on both the volume and the concentration of nutrients in agricultural drainage and runoff waters. Surface transport primarily consists of runoff, which involves interactions of water with nutrients on/near the soil surface, while sub-surface transport is dominated by leaching, including preferential flow through soil macropores and piston-type flow through micropores of the soil matrix^11^.

**Figure 1:**
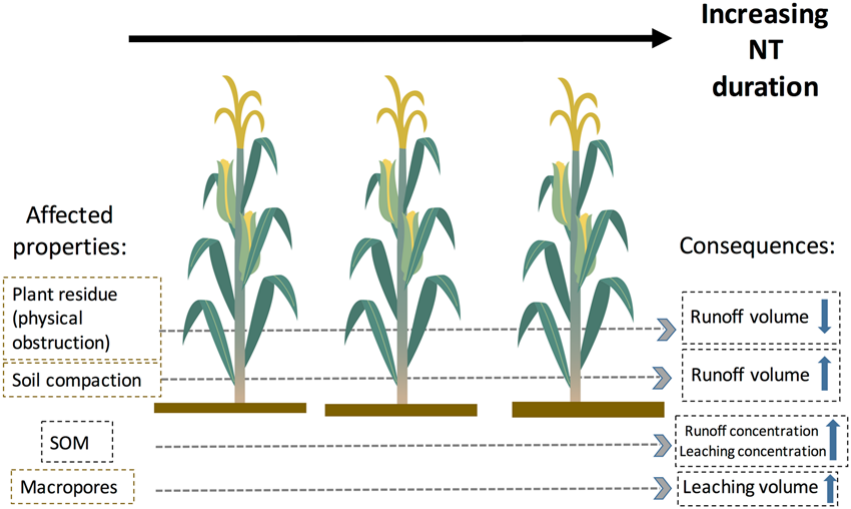
Diagram of NO_3_^−^ flow through leaching and runoff with NT management. Black arrow indicates an increase of NT duration from left to right. Dashed grey arrow indicates consequences of corresponding properties on the left to runoff and leaching volume and concentration. Blue arrow indicates an increase or decrease of runoff and leaching associated with each corresponding NT property on the left.

Since NT can differently impact NO_3_^−^ concentration and volumetric water flow, the net effect of NT on NO_3_^−^ load can be highly variable and dependent on the transport pathway considered^12^. While several studies have documented positive effects of NT in reducing NO_3_^−^ concentration in groundwater^4,13^, other studies have found no effect^14,15^. Many variables, including physical (e.g., rainfall variability, soil texture) and management factors (e.g., crop species, fertilizer type, tillage duration) likely affect NO_3_^−^ mobility and export from agricultural fields^16^. Changes in rainfall intensity, in particular, can influence the amount of NO_3_^−^ carried into the waterways as they affect the amount of water leaving the system^10^. Studies have reported no effect^10^, reduction^3^ or increase^17^ of NO_3_^−^ load with NT adoption. Other variabilities likely reflect the interactions of tillage practices with soil texture, crop type, and NT duration. For example, NO_3_^−^ load is expected to increase in sandy soils due the low NO_3_^−^ retention capacity in coarse soils^18^. Similarly, due to higher N fertilizer application rate, corn is likely to generate higher NO_3_^−^ load than crops such as soybean or alfalfa^3,4^. The effects of NT can be further affected by NT duration given the impact of long-term NT on SOM accrual. Improved soil biology and aeration with NT is a gradual process (Fig. 1), and their effects on N cycling processes (e.g., nitrification, denitrification and N immobilization) will only be manifested after certain period of NT implementation^19^. While conservation tillage has been shown to reduce runoff (reduction rate: by 15 to 89%^8^), the development of numerous macropores in NT soils could enhance NO_3_^−^ leaching^11^.

Partially due to the factors discussed above, the literature reviewed herein suggested that there has been no consensus on the effects of NT on NO_3_^−^ loss from agricultural fields. In this meta-analysis, we aimed to: (i) present a comprehensive comparison between NT and CT management with regard to NO_3_^−^ concentration and load, and (ii) explore how NO_3_^−^ loss (leaching and runoff) under NT co-vary with physical (i.e., aridity, rainfall variability, soil texture) and management factors (i.e., crop species, duration of tillage, fertilizer type). Based on available data and current understanding on the soil-plant relationships under NT, we expect that NT management (compared with CT) will generate higher NO_3_^−^ load through leaching due to greater abundance of macropores, but lower NO_3_^−^ loss through runoff. Ultimately, the comparison between these management practices depends on trade-offs between runoff volume and concentration (Fig. 1). However, since the mechanisms controlling NO_3_^−^ transport depend on pedogenetic processes and soil properties that evolve with time, one can expect the effect of NT in reducing NO_3_^−^ load to be site-specific and co-vary with other management variables including tillage duration.

## Results

**Figure 2:**
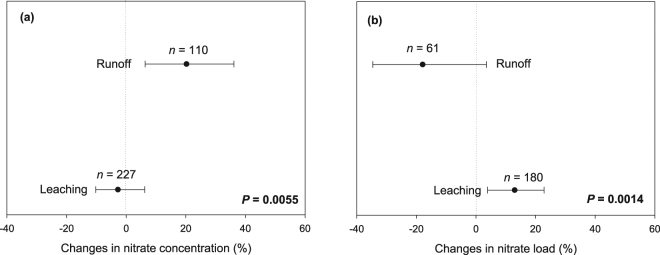
The overall percentage change in the concentration (**a**) and load (**b**) of nitrate with no-till (NT) in comparison to conventional tillage (CT). Black dots represent the mean of *lnR* with error bar representing the 95% confidence interval (CI). A negative value indicates a reduction due to NT adoption in comparison to CT, which is only statistically significant when the CI does not overlap zero. Letter ‘*n*’ indicates the number of samples, *P* values indicate statistical difference between leaching and runoff.

When comparing between NT and CT management, we found that NT provided no overall reduction in NO_3_^−^ concentration (Fig. 2a) or load (Fig. 2b). Although NT increased NO_3_^−^ runoff concentration, NO_3_^−^ runoff load was similar between NT and CT (i.e., confidence interval or CI overlaps zero; Fig. 2). In contrast, NT increased NO_3_^−^ load through leaching, despite generating similar NO_3_^−^ leachate concentration compared to CT (i.e., CI overlaps zero; Fig. 2). Therefore, leaching is the major pathway that contributes to ineffective control of NO_3_^−^ loss from agricultural fields managed under NT. Our analysis further revealed several physical and management variables affecting the extent of NO_3_^−^ loss via runoff and leaching transport pathways.

### Physical variables

**Figure 3:**
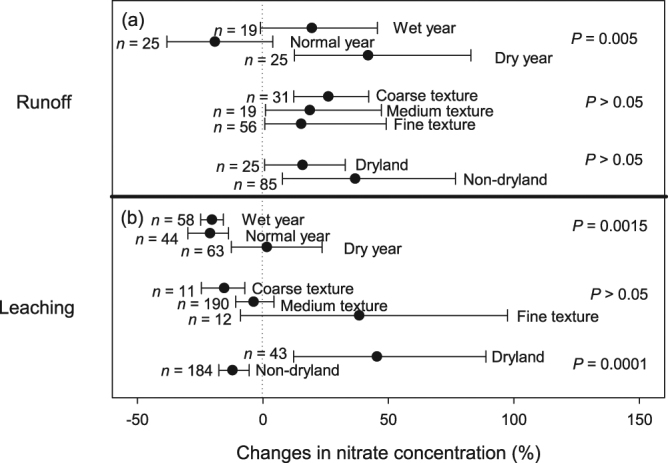
Percentage changes in the concentration of nitrate through runoff (**a**) and leaching (**b**) and their interactions with different physical variables. Black dots represent the mean of *lnR* with error bar representing the 95% confidence interval (CI). A negative value indicates a reduction due to NT adoption in comparison to CT, which is only statistically significant when the CI does not overlap zero. Letter ‘*n*’ indicates the number of sample, *P* values indicate difference within each physical variables.

In comparison to CT, NT generated higher runoff NO_3_^−^ concentration during dry years (Fig. 3a). This trend was consistent with the overall results of the meta-analysis (Fig. 2a), and increasing runoff NO_3_^−^ concentration was observed across different soil textures and eco-regions or aridity (Fig. 3a). During normal and wet years, NT and CT produced similar runoff NO_3_^−^ concentration (i.e., CI overlaps zero; Fig. 3a). However, NT reduced leachate NO_3_^−^ concentration in coarse-textured soils, during normal and wet years, and in the non-dryland regions (Fig. 3b). These leachate NO_3_^−^ concentration results were different from the overall trend of similar leachate NO_3_^−^ concentration under NT and CT (Fig. 2a).

**Figure 4:**
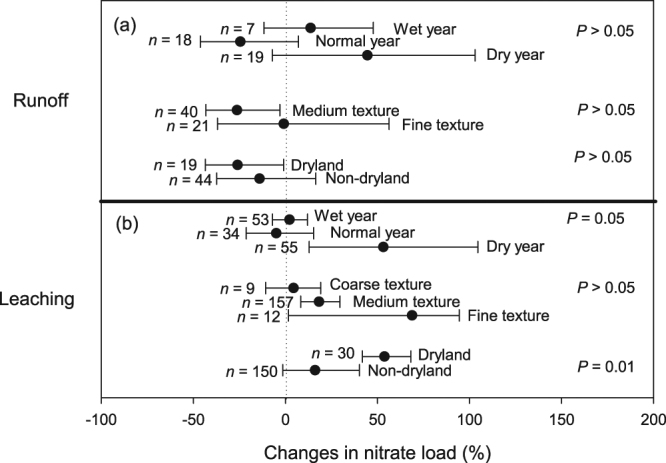
Percentage changes in the load of nitrate through runoff (**a**) and leaching (**b**) and their interactions with different physical variables. Black dots represent the mean of *lnR* with error bar representing the 95% confidence interval (CI). A negative value indicates a reduction due to NT adoption in comparison to CT, which is only statistically significant when the CI does not overlap zero. Letter ‘*n*’ indicates the number of sample, *P* values indicate difference within each physical variables.

In terms of load, NT was effective in reducing NO_3_^−^ load from runoff in the drylands and in medium-textured soils, although the differences within eco-region (drylands vs non-drylands) and soil texture category (medium vs fine soil texture) were not significant (Fig. 4a). NT also did not increase NO_3_^−^ loss via leaching in the non-dryland regions, during wet and normal years, as well as in coarse-textured soils (i.e., CI overlaps zero; Fig. 4b). NT, however, increased NO_3_^−^ loss in the drylands, during dry years and in medium- and fine-textured soils (Fig. 4b), consistent with the overall results of the meta-analysis (Fig. 2b).

### Management variables

**Figure 5:**
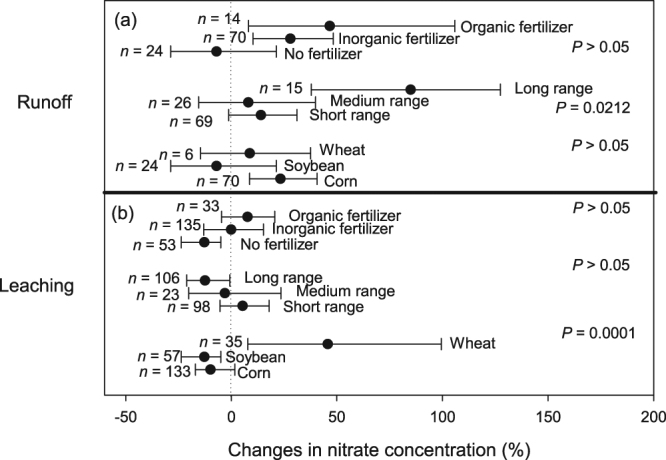
Percentage changes in the concentration of nitrate through runoff (**a**) and leaching (**b**) and their interactions with different management variables. Black dots represent the mean of *lnR* with error bar representing the 95% confidence interval (CI). A negative value indicates a reduction due to NT adoption in comparison to CT, which is only statistically significant when the CI does not overlap zero. Letter ‘*n*’ indicates the number of sample, *P* values indicate difference within each management variables.

Compared with CT, NO_3_^−^ concentration in runoff was higher with long-range NT duration, and when NT was combined with organic/inorganic fertilizer use and corn cultivation (Fig. 5a). Similar NO_3_^−^ concentration in runoff between NT and CT was mostly observed with short- to medium-range NT duration, when no N fertilizer was applied, and in wheat or soybean fields (i.e., CI overlaps zero; Fig. 5a). In contrast, reduction in leachate NO_3_^−^ concentration with NT was noted with long-range NT duration (>10 years), as well as in soybean or unfertilized fields (Fig. 5b), although the difference was only significant for crop species category (i.e., soybean and corn fields produced lower leachate NO_3_^−^ concentration than wheat; Fig. 5b). Interestingly, the influence of fertilizer type in determining the concentration of NO_3_^−^ through both runoff and leaching was not significant (Fig. 5).

**Figure 6:**
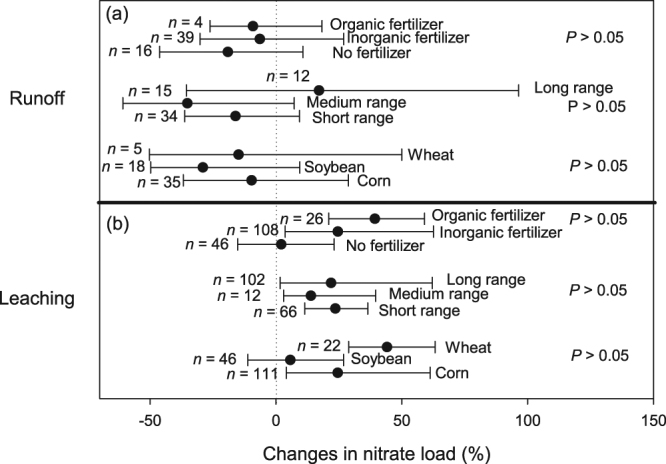
Percentage changes in the load of nitrate through runoff (**a**) and leaching (**b**) and their interactions with different management variables. Black dots represent the mean of *lnR* with error bar representing the 95% confidence interval (CI). A negative value indicates a reduction due to NT adoption in comparison to CT, which is only statistically significant when the CI does not overlap zero. Letter ‘*n*’ indicates the number of sample, *P* values indicate difference within each management variables.

In terms of NO_3_^−^ load through runoff, NT produced similar runoff load to CT, regardless of the management variables (i.e., all CIs overlap zero); (Fig. 6a). NT also increased leaching NO_3_^−^ loss compared to CT, regardless of NT duration, whether in wheat or corn fields as well as whether in fertilized soils (Fig. 6b). These findings were consistent with the overall trend of the meta-analysis (Fig. 2b). NT only produced similar NO_3_^−^ load to CT through leaching when NT was combined with soybean cultivation (no N fertilizer) (Fig. 6b).

## Discussion

Higher NO_3_^−^ concentration in runoff from NT than CT fields (Fig. 2a) likely reflects the difference in SOM quantity and the larger pool of nutrients in the surface layers of NT than CT soils^20^. However, it is important to note that in well-drained soils (indicated by the absence of artificial drainage), we found: (i) no difference between NT and CT with regard to runoff NO_3_^−^ concentration, and (ii) a reduction in NO_3_^−^ runoff load with NT. These discrepancies suggest that drainage characteristics could influence runoff and leaching processes, and ultimately the fate of NO_3_^−^ under NT (Supplementary Fig. S1). In well-drained soils, applied fertilizer N could be distributed more uniformly to a slightly deeper layer (as opposed to surface accumulation in poorly-drained and clay-rich soils)^21^. This could lead to a lower NO_3_^−^ concentration in runoff from well-drained soils. Here we suggest that the combination between surface compaction, drainage and clay content as a controlling factor of NO_3_^−^ concentration and load via leaching and runoff. Given the surface accumulation of nutrients and soil compaction under NT, in addition to surface sealing that often occur in clay-rich soils^22^, it is unsurprising that elevated runoff NO_3_^−^ concentration and load were recorded in these soils when compared to well-drained soils (Fig. 2 and Supplementary Fig. S1).

We should also highlight the fact that higher NO_3_^−^ concentration does not always translate into higher NO_3_^−^ load, particularly because load is also dependent on water volume^10,12^. Crop residue cover serves as a physical barrier in reducing the amount of water moving horizontally (runoff) under NT^23,24^. Restricted horizontal water movement and the built-up of macropores under NT allow more water to infiltrate^23^, which might explain why under NT runoff NO_3_^−^ load was lower than leachate NO_3_^−^ load (Fig. 2b). While the observed trend of increased NO_3_^−^ leachate load with NT is in accord with our hypothesis (i.e., higher leaching loss likely due to greater abundance of macropores and better soil infiltrability; Fig. 1), the contributing mechanism would primarily be an increase in leachate volume rather than NO_3_^−^ concentration. Such an increase in volumetric leachate amount would also explain the higher NO_3_^−^ leachate load (compared to NO_3_^−^ leachate concentration) observed under NT across different rainfall conditions, soil texture and aridity regimes (Figs 3b and 4b). For example, during dry years, NO_3_^−^ leachate concentration was similar under NT and CT, but NO_3_^−^ load was significantly higher under NT. Likewise, in the non-dryland regions, lower NO_3_^−^ leachate concentration under NT was accompanied by higher (although not significant) NO_3_^−^ load under NT than CT (i.e., confidence interval overlaps zero; Figs 3b and 4b). Thus, deviation between NT and CT was consistently greater when comparison is made on the basis of load (instead of concentration).

Overall, we found that the adoption of NT resulted in increased NO_3_^−^ loss via leaching compared to CT management (Fig. 2b). These results can be ascribed to the frequent occurrence of macropores (dead roots, earthworm burrows) in soils under long-range NT duration^11^. In addition to these preferential flow channels, an overall improvement in soil infiltration capacity (a consequence of SOM build-up and structure stability)^25^ under NT also contributes to higher water flux and increased NO_3_^−^ load through leaching. The loss of NO_3_^−^ can be further exacerbated by the presence of artificial sub-surface drainage systems (e.g., tiles) that are often installed in poorly-drained and clay-rich soils. Tile drainage increases the speed with which water moves off the landscape, thus short-cutting the natural water flow through the soil matrix^26,27^. Within this general trend, specific effects of physical factors and management variables on the results are discussed below.

The meta-analysis revealed indirect effects of soil texture in determining the impact of NT on NO_3_^−^ availability and transport in agroecosystems. In that regard, the reduction of runoff NO_3_^−^ load in soils of medium texture (Fig. 4a) and the reduction in leachate NO_3_^−^ concentration in coarse-textured soils with NT adoption are noteworthy observations (Fig. 3b). These observations can be associated with improvement in NO_3_^−^ retention in the soil matrix and/or better NO_3_^−^ utilization by crops under NT. NT is known to increase SOM content which, in turn, could translate into improved water availability for plant growth and better N use efficiency, particularly in sandy soils which naturally have low water holding capacity^28^. Similarly, NT was effective in reducing runoff NO_3_^−^ concentration in medium-textured but not in fine-textured soils, likely due to better water infiltrability with increased SOM and the absence of surface sealing that is often observed in clay soils^22^.

We also found that NT was effective in reducing runoff NO_3_^−^ load in the drylands, but generated similar or even higher NO_3_^−^ load than CT in most other cases (Fig. 4a). Due to the low amount of precipitation in the drylands, the volume of water that could be lost through runoff is necessarily low. The presence of physical barriers (surface crop residue) further contributes to the reduced NO_3_^−^ load observed in this eco-region despite elevated NO_3_^−^ concentration under NT (Figs 3a and 4a). However, in the drylands and during dry years (compared to non-drylands and normal/wet years), NT led to higher leachate NO_3_^−^ concentration than CT (Fig. 3b). Better soil moisture retention in NT than CT soils could lead to higher N mineralization. However, in these water-limited environments (dryland or dry years), plant growth and N uptake could become restricted, and that could lead to accumulation of soil mineral N. These residual mineral N pools can be mobilized during subsequent rainfall events, eventually leading to high NO_3_^−^ load (Fig. 4b). Taken together, these results (Figs 3b and 4b) therefore suggest that the aggravating effect of NT on NO_3_^−^ leaching loss likely involves an overall reduction of plant N uptake during dry conditions but an increase in soil N mineralization in NT than CT soils^29^. Partly due to these aforementioned processes and the complexity of plant-soil interactions, it is unsurprising that the net effect of NT in reducing NO_3_^−^ loss is sometimes difficult to demonstrate.

Although not always statistically significant, the effect of crop species also stands out. In soybean fields, leachate NO_3_^−^ concentration was lower under NT (Fig. 5b) and NO_3_^−^ load via leaching was similar under NT and CT (Fig. 6b). In contrast, in fields planted with wheat and corn, no beneficial effect of NT on NO_3_^−^ loss was observed (Fig. 6b). These observations suggest that leaching NO_3_^−^ loss under NT can be curtailed by reducing N fertilizer application rates. However, a reduction in synthetic fertilizer application rate would require further studies since this strategy could result in decreased crop yield, and therefore not acceptable to farmers. Alternative N management practices such as application of slow-release N fertilizer formulations^30^, injection and deep placement of fertilizer^31^ have shown significant promises, and deserve further investigations. In particular, research has shown that some cover crops can provide at least part of the mineral N needed for optimum crop yield, leading to possible reductions in the amount of synthetic N fertilizer applied to agricultural fields. In addition, slow-degrading cover crop plant materials such as rye (Secale cereale L.) release mineral N in synchrony with N demand of growing crops and thus enhance N uptake^32^. These results argue for the supplementation of NT farming with other strategies to enhance N use efficiency and reduce diffuse N pollution.

Higher leachate NO_3_^−^ concentration in fields cultivated with wheat compared to those planted to corn (Fig. 5b) was unexpected because corn usually requires higher N fertilizer rate (~200 kg N ha^−1^)^33^ than wheat (~45 kg N ha^−1^)^34^. These intriguing results could be due to the time gap between fertilizer application to wheat and the growing period of that crop. About 65% of our data came from winter wheat cultivation in which fertilizer application generally occurs prior to wheat planting^35^. Following winter wheat dormancy, elevated concentration of residual soil NO_3_^−^ has been reported and, upon thawing, this residual NO_3_^−^ tends to move from the surface to the deeper soil layers with snow-melt water^35^. Taken together, these processes could contribute to the higher leachate NO_3_^−^ concentration with that crop.

The observed greater NO_3_^−^ concentration in runoff from fields under long-range than short-range NT duration (Fig. 5a) is most likely linked to deposition of crop residue and SOM accumulation with time on NT soil surface, consistent with our hypothesis (Fig. 1), and as reported in other studies^20,36^. In contrast, the effect of NT duration on leaching NO_3_^−^ loss is more complex to interpret due to the divergent impact of that practice on NO_3_^−^ concentration and water flux. It has been shown that long-range NT duration can lead to improved plant-soil interactions and better N retention, including immobilization in the microbial biomass^29^. These processes may have contributed to the observed reduction in leachate NO_3_^−^ concentration with long-range NT duration (Fig. 5b). However, this reduction in concentration does not necessarily translate into a reduction in load due to increased vertical water flux under NT (Fig. 6b). Under long-range NT duration, crop residue accumulates on soil surface, acts as physical barrier to runoff, and thereby allows more water to infiltrate into the soil. The development of macropores further facilitates the vertical water flux at medium- to long-range NT sites^11^. This interpretation is consistent with the significantly higher NO_3_^−^ load through leaching observed under long-range than under short-range NT duration (Supplementary Fig. S5).

## Conclusions

Our analysis shows that NT farming generally result in increased NO_3_^−^ loss, with the exception of some specific physical and management conditions under which reduction in NO_3_^−^ load was observed. These NO_3_^−^ load reductions were likely associated with a reduction in surface runoff volume under NT. Since NT has a pronounced effect on the distribution of crop residue and nutrients, occasional soil harrowing (i.e., once in 10 or more years) may help overcome some of the soil compaction and nutrient stratification problems that are often associated with NT, particularly in fine-textured soils, without causing significant loss of organic matter and deterioration of soil structure^22^. This intervention may also cause disturbance of macropores continuity, resulting in reduced transport capacity of macropres and their significance as major pathways for NO_3_^−^ loss in NT systems. We also suggest that NT be combined with other land management practices (e.g., injection of fertilizer, cover cropping, intercropping or rotation with perennial crops) to improve N use efficiency and reduce NO_3_^−^ loss from agricultural fields.

## Methods

Peer-reviewed journal articles published in English from 1985 to 2016 were collected to build the database using the Web of Science search platform and the following sets of topic keywords: (i) tillage or plow or plough, (ii) nitrate or water quality, and (iii) soybean or corn or wheat. We selected those three crops based on the 2012 FAO’s Crop Production Statistics^37^ and the understanding that these crops are likely to be cultivated using NT and CT practices. Due to the variability of tillage methods, conventional tillage (CT) was broadly defined to encompass all forms of tillage (e.g., moldboard, rotary, chisel and disking), while NT farming was taken as synonymous to zero tillage. Of the 1688 articles found, only articles that reported the concentration and/or load of NO_3_^−^ in paired NT vs CT practices under field conditions, including lysimeter studies, were included in the database. The data were recorded separately for NO_3_^−^ concentration and load, and the magnitude of each was then examined based on the major pathways of NO_3_^−^ loss, namely surface runoff and leaching. To ensure that we captured the actual NO_3_^−^ loss, we did not consider soil NO_3_^−^ as NO_3_^−^ loss. Therefore, only NO_3_^−^ concentration measured in tile drains, leachate, groundwater and lysimeters was used as a proxy for NO_3_^−^ leachate concentration. Surface runoff NO_3_^−^ was defined as NO_3_^−^ that can be sourced to the surface soil layers (not from groundwater, tile drainage, or leachate) and transferred to surface water bodies. Since our focus was on understanding the effects of NT practice (both short-range and long-range duration) on NO_3_^−^ loss, we did not include articles that described one-time tillage experiments on land previously under NT as this could induce artifacts. The list of the articles used for this study is provided as Supplementary Table S1.

To further disentangle the effects of other co-varying factors on NO_3_^−^ loss via surface runoff or leaching, the data were further analysed using the following two major categorical variables (i.e., physical and management categorical variables), except when there were constraints of data availability. Physical categorical variables include: (i) rainfall variability (i.e., wet, normal, and dry years), (ii) aridity (dryland and non-dryland eco-regions), and (iii) soil texture (fine-, medium-, or coarse-textured soil). Management categorical variables include: (i) crop species (i.e., wheat, soybean, or corn), (ii) tillage duration (i.e., short, medium, and long-range), and (iii) fertilizer type (i.e., no fertilizer, organic, inorganic). Those categories were selected because they were the most commonly noted in published reports and most widely available in the agronomic literature. We used aridity index (AI) for differentiating between dryland and non-dryland regions. Areas with AI < 0.65 are considered as drylands^38^. For differentiating among soil textural classes, we used the United States Department of Agriculture (USDA) soil texture triangle, and considered clay, sandy-clay, and silty-clay soils as fine texture; silt, silt-loam, silty-clay-loam, loam, sandy clay-loam soils, and clay-loam soils as medium texture; and sand, loamy-sand, and sandy-loam as coarse texture^39^. We considered short-range NT if the practice was adopted for <5 years, medium-range if it was in place for 6–10 years, and long-range if the practice was applied for >10 years^40^. The duration of the tillage practice was calculated as the length between the start of NT treatment and the time of observation. For the purposes of this meta-analysis, we established discrete levels for each variable and coded each observation accordingly (Supplementary Table S2).

Due to variations in land cultivation practices, there were other criteria imposed when calculating the effect of NT for each category. For example, in calculating the effect of crop species, we were unable to quantify the effects of crop rotation because the load or concentration of NO_3_^−^ was generally observed annually or during a growing season. The effects of rotation, on the other hand, involved different crop species and spanned over a longer period of time. In accord with that line of reasoning, data points that averaged NO_3_^−^ concentration or load across multiple years could not be included in determining the effects of crop species since these data points may include several crop species. In addition, only studies involving a single crop (i.e., not intercropping) were included in calculating the co-varying effect of crop species. Similar criteria were also imposed when assessing the effect of rainfall variability. Since we were interested in differentiating tillage behaviour during dry and wet years, we recorded the amount of rainfall for each year (or growing season) of observation reported in the study when evaluating the effect of rainfall variability. Therefore, we did not include any studies that only reported the average amount of nutrient loss across multiple years when evaluating the effect of rainfall variability since these average values did not reflect possible changes in nutrient loss under varying rainfall distribution. These rainfall values were compared to long-term average for the region, and we used a simple definition of “dry year” based on 10% rainfall deficiency^41^. A similar deviation (surplus) was applied to define “wet year”. We also did not include studies that involved rainfall simulation or irrigation when calculating the effects of rainfall variability.

We applied a rigorous procedure to ensure the independence of each data entry, avoiding over-representation of any particular study and reducing publication bias^42^. For example, if leaching observations were made at several depths in a study, we averaged the response of each depth and only a single data entry was used in the meta-analysis. Similarly, if a study reported different sampling times (e.g., monthly or weekly, certain phenological phases), the response was averaged, and only one sampling time (i.e., the annual or growing season) for the corresponding year or growing season was used in the meta-analysis. However, if a study examined the effect of tillage in combination with other agronomic factors (e.g., fertilizer type or tillage method), the data points were treated as separate observations^42^. Similarly, if a study was conducted in different years or locations, the data were treated separately since a given field could have experienced different rainfall variability or have been planted to different crops over the years. We, however, did not differentiate between the timing of the observation (e.g., annual or growing season) when evaluating the effects of NT as the impact of NT on water quality is expected to extend beyond the sampling time.

The nutrient load or concentration ratio between NT and CT fields (instead of actual NO_3_^−^ load or concentration) was used. Since we used ratio, one paired site with three years of annual measurement, for example, would correspond to three data points, instead of six. The total number of data points was 337 from 43 studies, and 241 from 33 studies for NO_3_^−^ concentration and load, respectively. To avoid the potential bias from artificial sub-surface drainage (e.g., tile drainage), data from sites without tile drainage were analysed separately (these results are provided in Supplementary Figs S1–S5). While this separation allowed us to tease out each of the co-varying factors that could affect NO_3_^−^ loss via artificial drainage, it should be noted that: (i) there were some unrepresented categories due to constraint of data availability, and (ii) some categories represented in fewer studies than others, and the explanatory power of these categorical variables could be limited.

To quantify the difference in NO_3_^−^ concentration and load due to NT, meta-analysis was used to construct the confidence intervals for each of the aforementioned categorical variables. The response ratio (*R*) is defined as the ratio between the outcome of experimental group (i.e., NT) to that of the control group (i.e., CT) to estimate the proportional changes resulting from tillage removal. The use of ratio also minimized the variability that occurred across different management strategies but could not be captured into certain categories due to limited data availability. Since only 12 out of 43 studies reported the standard deviation, we performed an unweighted analysis using the log response ratio (*lnR*) to calculate bootstrapped confidence limits using the statistical software MetaWin 2.0^43^ in order to include the majority of studies that did not report sample size or standard deviation^44^. To improve the reliability of *lnR* in estimating the effect size of the response ratio, we performed a diagnostic test using the formula:

$$\frac{x}{SD}(\frac{4{n}^{\frac{3}{2}}}{1+4n})\ge 3$$

where *x* is the mean, *SD* is the standard deviation and *n* is the sample size^45^. The results of this calculation are provided in Supplementary Table S3. Bootstrapping was also iterated 9999 times to improve the probability that the confidence interval was calculated around the cumulative mean effect size for each categorical variable. The sample size (n) of each bootstrapping are reported in each figure. The difference between NT and CT treatment is considered statistically significant if the 95% confidence interval (CI) does not overlap zero, while the difference between categorical variables is considered significant if the bootstrap CI does not overlap each other^42,46^. Statistical significance was determined at *P* < 0.05.

## Electronic supplementary material

Supplementary Information
