# Supplementary material for: Impacts of no-tillage management on nitrate loss from corn, soybean and wheat cultivation: A meta-analysis
Source: Sci Rep. 2017 Sep 21;7:12117. doi: 10.1038/s41598-017-12383-7 (PMC5608951; doi:10.1038/s41598-017-12383-7)
Supplement: Supplementary file 1 — Supplementary Information [file 41598_2017_12383_MOESM1_ESM.pdf]

Impacts of no-tillage management on nitrate loss from corn, soybean and wheat cultivation: A meta-analysis

Stefani Daryanto[1], Lixin Wang[1]*, Pierre-André Jacinthe[1]

[1]Department of Earth Sciences, 723 West Michigan St, SL118, Indiana University Purdue University Indianapolis, Indianapolis, IN 46202, USA

*Corresponding Author: Lixin Wang (lxwang@iupui.edu)

Supplementary Figures

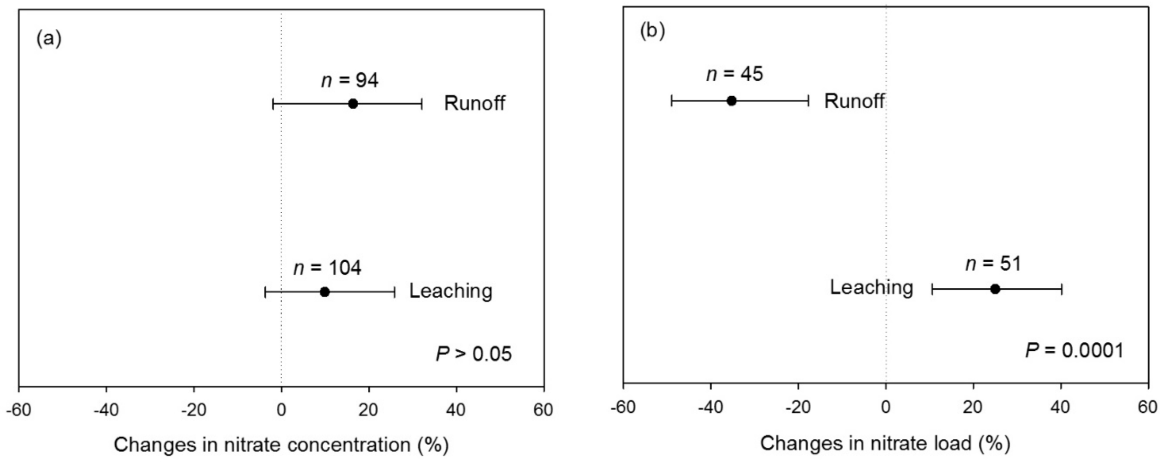

Fig. S1. The overall percentage change in the concentration (a) and load (b) of nitrate with no-till (NT) in comparison to conventional tillage (CT) from soils without artificial drainage. Black dots represent the mean of *lnR* with error bar representing the 95% confidence interval (CI). A negative value indicates a reduction due to NT adoption in comparison to CT, which is only statistically significant when the CI does not overlap zero. Letter '*n*' indicates the number of samples, *P* values indicate statistical difference between leaching and runoff.

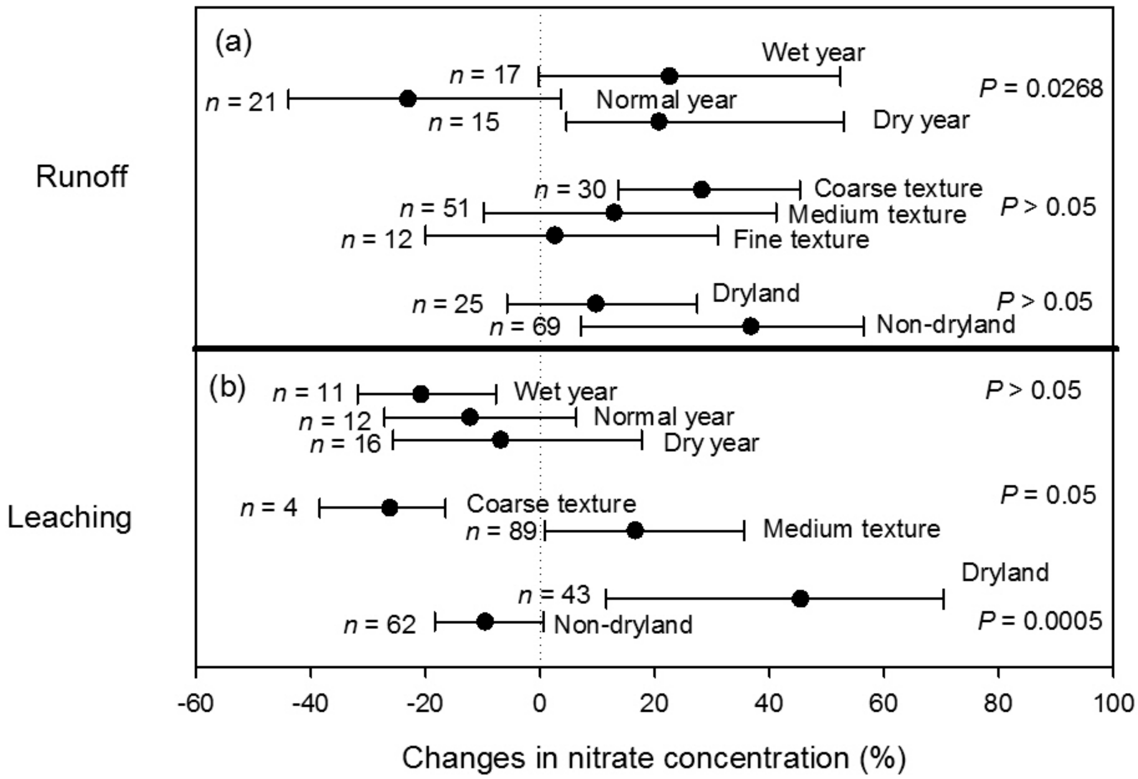

Fig. S2. Percentage changes in the concentration of nitrate through runoff (a) and leaching (b) and their interactions with different physical variables from soils without artificial drainage. Black dots represent the mean of *lnR* with error bar representing the 95% confidence interval (CI). A negative value indicates a reduction due to NT adoption in comparison to CT, which is only statistically significant when the CI does not overlap zero. Letter '*n*' indicates the number of sample, *P* values indicate difference within each physical variables.

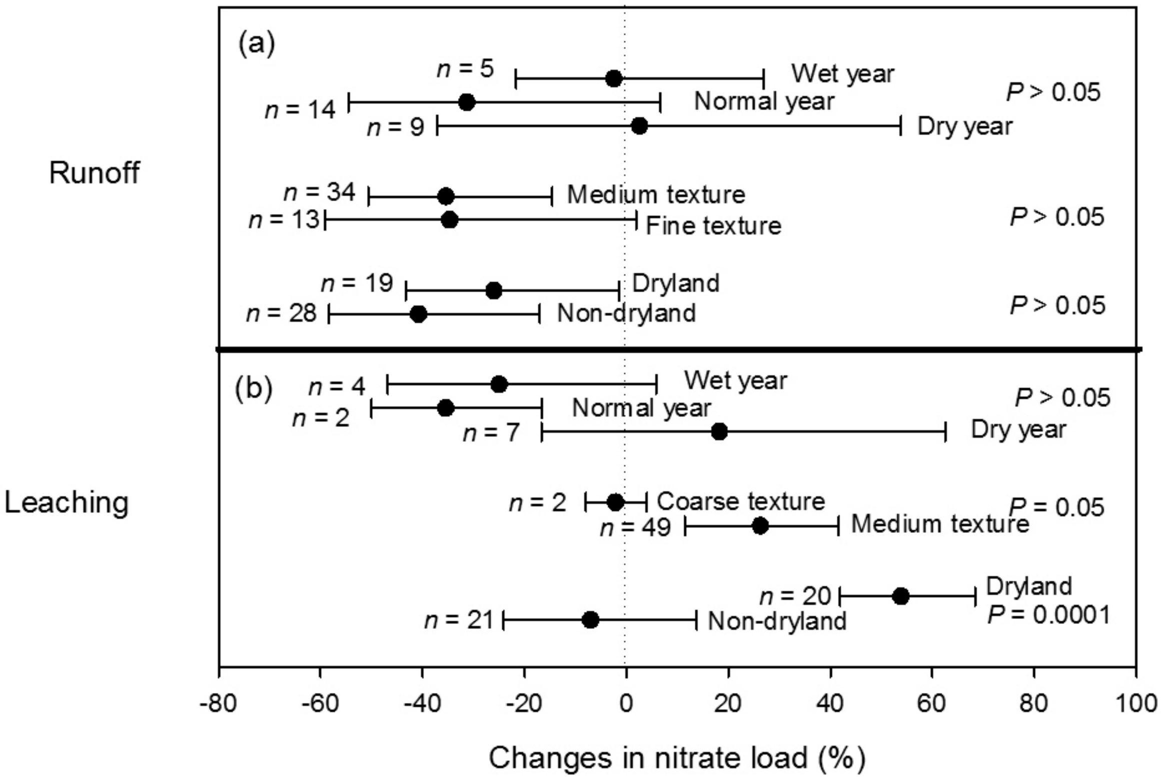

Fig. S3. Percentage changes in the load of nitrate through runoff (a) and leaching (b) and their interactions with different physical variables from soils without artificial drainage. Black dots represent the mean of *lnR* with error bar representing the 95% confidence interval (CI). A negative value indicates a reduction due to NT adoption in comparison to CT, which is only statistically significant when the CI does not overlap zero. Letter '*n*' indicates the number of sample, *P* values indicate difference within each physical variables.

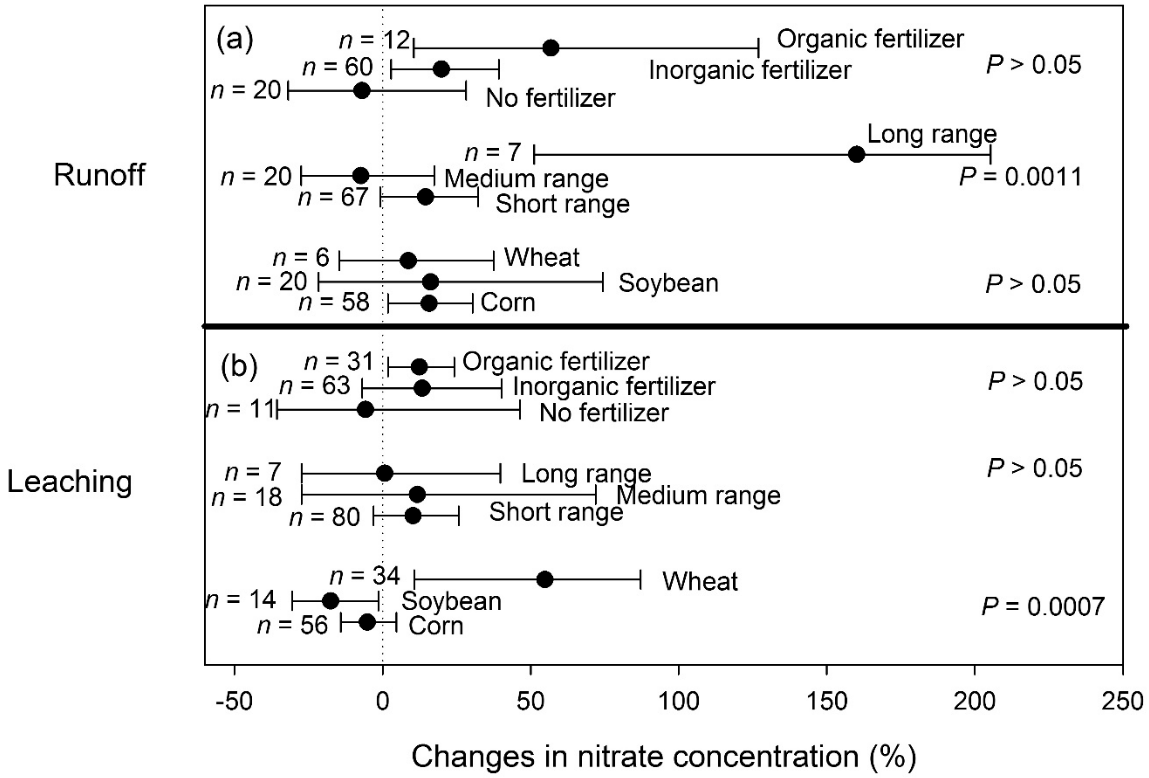

Fig. S4. Percentage changes in the concentration of nitrate through runoff (a) and leaching (b) and their interactions with different management variables from soils without artificial drainage. Black dots represent the mean of *lnR* with error bar representing the 95% confidence interval (CI). A negative value indicates a reduction due to NT adoption in comparison to CT, which is only statistically significant when the CI does not overlap zero. Letter '*n*' indicates the number of sample, *P* values indicate difference within each management variables.

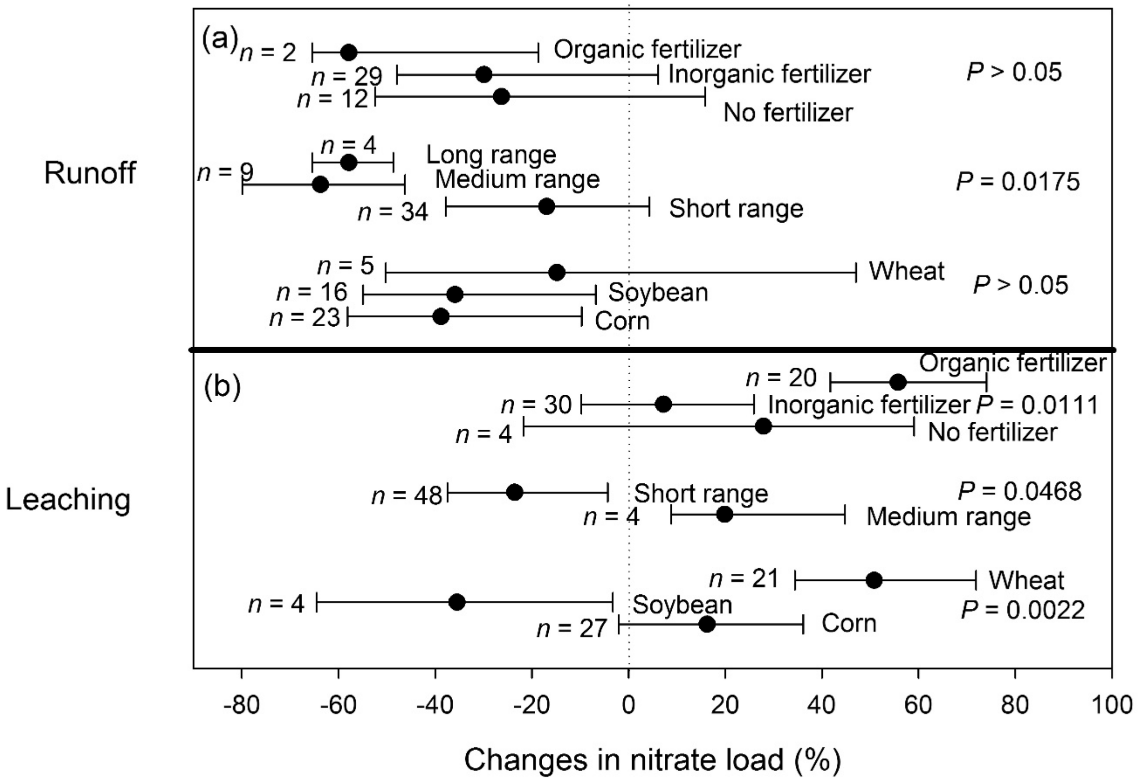

Fig. S5. Percentage changes in the load of nitrate through runoff (a) and leaching (b) and their interactions with different management variables from soils without artificial drainage. Black dots represent the mean of *lnR* with error bar representing the 95% confidence interval (CI). A negative value indicates a reduction due to NT adoption in comparison to CT, which is only statistically significant when the CI does not overlap zero. Letter ‘*n*’ indicates the number of sample, *P* values indicate difference within each management variables.

Supplementary Table S1. List of publications used to collect the database for this study

Author and year of publication	Article Title and Journal name	Nitrate concentration	Nitrate load
Al-Kaisi and Licht, 2004	Effect of strip tillage on corn nitrogen uptake and residual soil nitrate accumulation compared with no-tillage and chisel plow. Agronomy Journal 96, 1164-1171	X*	
Bakhsh and Kanwar, 2001	Simulating tillage effects on non-point source pollution from agricultural lands using GLEAMS. Transactions of the ASAE 44, 891-898	X	X
Bakhsh et al., 2002	Cropping system effects on NO_3–N loss with subsurface drainage water. Transactions of the ASAE 45, 1789-1797	X	X
Bakhsh and Kanwar, 2007	Tillage and N application rates affect on corn and soybean yields and NO_3-N leaching losses. Transactions of the ASABE 50, 1189-1198	X	X
Bjorneberg et al., 1996	Seasonal changes in flow and nitrate-N loss from subsurface drains. Transactions of the ASAE 39, 961-976	X	X
Blevins et al., 1990	Tillage effects on sediment and soluble nutrient losses from a maury silt loam soil. Journal of Environmental Quality 19, 683-686		X
Brye et al., 2001	Nitrogen and carbon leaching in agroecosystems and their role in denitrification potential. Journal of Environmental Quality 30, 1822-1828	X	X
Brye and Norman, 2004	Land-use effects on anion-associated cation leaching in response to above normal precipitation. Acta Hydrochimica et Hydrobiologica 32, 235-248	X	X
Chichester and Richardson, 1992	Sediment and nutrient loss from clay soils as affected by tillage. Journal of Environmental Quality 21, 587-590		X

Drury et al., 1993	Influence of tillage on nitrate loss in surface runoff and tile drainage. Soil Science Society of American Journal 57, 797-802	X	X
Eghball and Gilley, 1999	Phosphorus and nitrogen in runoff following beef cattle manure or compost application. Journal of Environmental Quality 28, 1201-1210	X	
Eghball et al., 2000	Narrow grass hedge effects on phosphorus and nitrogen in runoff following manure and fertilizer application. Journal of Soil and Water Conservation 55, 172-176	X	X
Fraser et al., 2013	Winter nitrate leaching under different tillage and winter cover crop management practices. Soil Science Society of American Journal 77, 1391-1401	X	
Fuller et al., 2010	Seasonal and crop rotational effects of manure management on nitrate–nitrogen leaching in Nova Scotia. Agriculture, Ecosystems and Environment 137, 267-275	X	X
Gerakis et al., 2006	Simulation of leaching losses in the nitrogen cycle. Communications in Soil Science and Plant Analysis 37, 1973-1997		X
Gilley et al., 2007	Nutrient concentrations of runoff during the year following manure application. Transactions of the ASABE 50, 1987-1999	X	
Gilley et al., 2007b	Nitrogen and phosphorus concentrations of runoff as affected by moldboard plowing. Transactions of the ASABE 50, 1543-1548	X	
Gilley et al., 2010	Nutrient transport in runoff as affected by diet, tillage and manure application rate. Transactions of the ASABE 53, 1895-1902		X
Huang et al., 2015	No-tillage and fertilization management on crop yields and nitrate leaching in North China Plain. Ecology and Evolution 5, 1143-1155	X	X

Joshi et al., 1994	Long-term conservation tillage and liquid dairy manure effects on corn. II. Nitrate concentration in soil water. Soil and Tillage Research 31, 225-233	X	
Kanwar et al., 1997	Ridge, moldboard, chisel, and no-till effects on tile water quality beneath two cropping systems. Journal of Production Agriculture 10, 227-234	X	X
Lal et al., 1997	Soil degradative effects of slope length and tillage methods on alfisols in western Nigeria. II. Soil chemical properties, plant nutrient loss and water quality. Land Degradation and Development 8, 221-244	X	
Logan et al., 1994	Tillage, crop and climatic effects on runoff and tile drainage losses of nitrate and four herbicides. Soil and Tillage Research 30, 75-103	X	X
Meek et al., 1995	Nitrate leaching under furrow irrigation as affected by crop sequence and tillage. Soil Science Society of American Journal 59, 204-210	X	
Meisinger et al., 2015	Effects of tillage practices on drainage and nitrate leaching from winter wheat in the Northern Atlantic Coastal-Plain USA. Soil and Tillage Research 151, 18-27	X	X
Mkhabela et al., 2008	Gaseous and leaching nitrogen losses from no-tillage and conventional tillage systems following surface application of cattle manure. Soil and Tillage Research 98, 187-199	X	X
Mostaghimi et al., 1992	Crop residue effects on nitrogen yield in water and sediment runoff from two tillage systems. Agriculture, Ecosystems and Environment 39, 187-196	X	
Owens and Edwards, 1993	Tillage studies with a corn-soybean rotation: Surface runoff chemistry. Soil Science Society of American Journal 57, 1055-1060	X	X

Patni et al., 1996	Tile effluent quality and chemical losses under conventional and no tillage .1. Flow and nitrate. Transactions of the ASAE 39, 1665-1672	X	X
Patni et al., 1998	Groundwater quality under conventional and no tillage: I. Nitrate, electrical conductivity, and pH. Journal of Environmental Quality 27, 869-877	X	X
Randall and Iragavarapu, 1995	Impact of long-term tillage systems for continuous corn on nitrate leaching to tile drainage. Journal of Environmental Quality 24, 360-366	X	X
Rasse and Smucker, 1999	Tillage effects on soil nitrogen and plant biomass in a corn-alfalfa rotation. Journal of Environmental Quality 28, 873-880	X	X
Richardson and King, 1995	Erosion and nutrient losses from zero tillage on a clay soil. Journal of Agricultural Engineering Research 61, 81-86		X
Ritter et al., 1993	Nitrate leaching under irrigated corn. Journal of Irrigation and Drainage Engineering 119, 544-553	X	X
Schreiber and Cullum, 1998	Tillage effects on surface and groundwater quality in loessial upland soybeanwatersheds. Transactions of the ASAE 41, 607-614	X	
Seta et al., 1993	Reducing soil erosion and agricultural chemical losses with conservation tillage. Journal of Environmental Quality 22, 661-665	X	X
Sharpley and Smith, 1994	Wheat tillage and water quality in the Southern Plains. Soil and Tillage Research 30, 33-48	X	
Sharpley et al., 2015	Arkansas discovery farms: Documenting water quality benefits of on-farm conservation management and empowering farmers. Acta Agriculturae Scandinavica, Section B—Soil & Plant Science 65, 186-198	X	
Singer et al., 2004	Tillage and compost affect yield of corn, soybean and wheat and soil	X	

	fertility. Agronomy Journal 96, 531-537		
Singh and Kanwar, 1995	Simulating NO3-N transport to subsurface drain flows as affected by tillage under continuous corn using modified RZWQM. Transactions of ASAE 38, 499-508	X	X
Smith et al., 1991	Water quality impacts associated with wheat culture in the southern plains. Journal of Environmental Quality 20, 244-249	X	X
Staver and Brinsfield, 1998	Using cereal grain winter cover crops to reduce groundwater nitrate contamination in the mid-Atlantic coastal plain. Journal of Soil and Water Conservation 53, 230-240	X	
Stoddard et al., 2005	Fertilizer, tillage, and dairy manure contributions to nitrate and herbicide leaching. Journal of Environmental Quality 34, 1354-1362	X	
Wang et al., 2015	Conservation tillage and optimized fertilization reduce winter runoff losses of nitrogen and phosphorus from farmland in the Chaohu Lake region, China. Nutrient Cycling in Agroecosytems 101, 93-106	X	X
Weed and Kanwar, 1996	Nitrate and water present in and flowing from root-zone soil. Journal of Environmental Quality 25, 709-719	X	X
Zeimen et al., 2006	Combining management practices to reduce sediment, nutrients and herbicides in runoff. Journal of Soil and Water Conservation 61, 258-267	X	X
Zhu et al., 2003	Tillage Effects on Nitrate Leaching Measured by Pan and Wick Lysimeters. Soil Science Society of American Journal 67, 1517-1523	X	X

*X mark indicates that data are available from the corresponding publication

Supplementary Table S2. Database of this study. Highlighted data indicate artificial drainage.

outlet types	outlet cate-gory	tillage durati on	tillage prac-tice	location	arid-ity	crop type	fertili -zer type	tex-ture class	rain-fall (mm)	Long-term mean rainfall (mm)	rainfall temporal resolution	Hydro-logical condi-tion	nitrate concen-tration NT (ppm)	nitrate concen-tration CT (ppm)	nitrate load NT (kg/ha)	nitrate load CT (kg/ha)	Reference
ground water	ground	short	mold board	maryland USA	non-arid	corn	inorg anic	N/A	1043	1118	annual	normal	12.25	14.75			staver & brinsfield
ground water	ground	short	mold board	maryland USA	non-arid	corn	inorg anic	N/A	837	1118	annual	dry	16.50	16.25			staver & brinsfield
ground water	ground	short	mold board	maryland USA	non-arid	corn	inorg anic	N/A	976	1118	annual	dry	17.63	16.1			staver & brinsfield
ground water	ground	short	mold board	maryland USA	non-arid	corn	inorg anic	N/A	1492	1118	annual	wet	19.00	22.25			staver & brinsfield
ground water	ground	med	mold board	maryland USA	non-arid	corn	inorg anic	N/A	1335	1118	annual	wet	9.50	17.125			staver & brinsfield
ground water	ground	med	mold board	maryland USA	non-arid	corn	inorg anic	N/A	954	1118	annual	dry	7.70	14.3			staver & brinsfield
ground water	ground	med	mold board	maryland USA	non-arid	corn	inorg anic	N/A	1010	1118	annual	normal	7.10	12.125			staver & brinsfield
ground water	ground	med	mold board	maryland USA	non-arid	corn	inorg anic	N/A	1097	1118	annual	normal	4.93	8.7			staver & brinsfield
ground water	ground	med	mold board	maryland USA	non-arid	corn	inorg anic	N/A	1338	1118	annual	wet	4.90	7			staver & brinsfield
ground water	ground	long	mold board	maryland USA	non-arid	corn	inorg anic	N/A	1072	1118	annual	normal	4.80	6.3			staver & brinsfield

ground water	ground	long	mold board	maryland USA	non-arid	corn	inorganic	N/A	1404	1118	annual	wet	3.60	4			staver & brinsfield
runoff	surface	med	mold board	ontario Canada	non-arid	corn	inorganic	medium	709	819	annual	dry	8.86	7.05	2.64	1.87	drury et al
runoff	surface	med	mold board	ontario Canada	non-arid	corn	inorganic	medium	827	819	annual	normal	5.56	4.04	4.1	2.51	drury et al
runoff	surface	med	mold board	ontario Canada	non-arid	corn	inorganic	medium	561	819	annual	dry	26.70	3.43	1.99	0.29	drury et al
runoff	surface	med	mold board	ontario Canada	non-arid	corn	inorganic	medium	709	819	annual	dry	8.86	6.86	2.64	2.62	drury et al
runoff	surface	med	mold board	ontario Canada	non-arid	corn	inorganic	medium	827	819	annual	normal	5.56	6.06	4.1	5.48	drury et al
runoff	surface	med	mold board	ontario Canada	non-arid	corn	inorganic	medium	561	819	annual	dry	26.70	12.4	1.99	1.7	drury et al
drain water	ground	med	mold board	ontario Canada	non-arid	corn	inorganic	medium	709	819	annual	dry	14.80	17.3	13.8	18	drury et al
drain water	ground	med	mold board	ontario Canada	non-arid	corn	inorganic	medium	709	819	annual	dry	14.80	15.5	13.8	14.7	drury et al
drain water	ground	med	mold board	ontario Canada	non-arid	corn	inorganic	medium	827	819	annual	normal	12.20	13	20	28.5	drury et al
drain water	ground	med	mold board	ontario Canada	non-arid	corn	inorganic	medium	827	819	annual	normal	12.20	12.3	20	19.8	drury et al
ground water	ground	short	mold board	mississipi USA	non-arid	soybean	none	medium	1783	1120	annual	wet	4.50	5.83			schreiber & cullum
ground water	ground	short	mold board	mississipi USA	non-arid	soybean	none	medium	1384	1120	annual	wet	4.09	8.06			schreiber & cullum

ground water	ground	short	mold board	mississipi USA	non-arid	soybean	none	medium	1126	1120	annual	normal	5.56	5.11			schreiber & cullum
runoff	surface	short	mold board	mississipi USA	non-arid	soybean	none	medium	1783	1120	annual	wet	0.64	0.53			schreiber & cullum
runoff	surface	short	mold board	mississipi USA	non-arid	soybean	none	medium	1384	1120	annual	wet	0.64	0.28			schreiber & cullum
runoff	surface	short	mold board	mississipi USA	non-arid	soybean	none	medium	1126	1120	annual	normal	0.89	0.79			schreiber & cullum
drain water	ground	long	mold board	iowa USA	non-arid	corn	inorganic	medium	1141	758	growing season	wet	39.00	64	96	52	kanwar et al; baksh & kanwar; singh & kanwar
drain water	ground	long	mold board	iowa USA	non-arid	corn	inorganic	medium	892	758	growing season	wet	19.00	34	56	56	kanwar et al; baksh & kanwar; singh & kanwar
drain water	ground	long	mold board	iowa USA	non-arid	corn	inorganic	medium	741	758	growing season	normal	11.00	16	18	17	kanwar et al; baksh & kanwar; singh & kanwar
drain water	ground	long	mold board	iowa USA	non-arid	corn	inorganic	medium	1141	758	growing season	wet	23.00	27	33	34	kanwar et al; baksh & kanwar; singh & kanwar
drain water	ground	long	mold board	iowa USA	non-arid	corn	inorganic	medium	892	758	growing season	wet	17.00	22	27	32	kanwar et al; baksh & kanwar; singh & kanwar
drain water	ground	long	mold board	iowa USA	non-arid	corn	inorganic	medium	741	758	growing season	normal	8.00	11	4	9	kanwar et al; baksh & kanwar; singh & kanwar

drain water	ground	long	mold board	iowa USA	non-arid	soybean	none	medium	1141	758	growing season	wet	22.00	37	33	37	kanwar et al; baksh & kanwar; singh & kanwar
drain water	ground	long	mold board	iowa USA	non-arid	soybean	none	medium	892	758	growing season	wet	11.00	15	29	38	kanwar et al; baksh & kanwar; singh & kanwar
drain water	ground	long	mold board	iowa USA	non-arid	soybean	none	medium	741	758	growing season	normal	9.00	12	4	11	kanwar et al; baksh & kanwar; singh & kanwar
drain water	ground	long	mold board	iowa USA	non-arid	corn	inorganic	medium	1050	758	growing season	wet			107.20	58.1	bakhsh & kanwar
drain water	ground	long	mold board	iowa USA	non-arid	corn	inorganic	medium	970	758	growing season	wet			63.00	62.7	bakhsh & kanwar
drain water	ground	long	mold board	iowa USA	non-arid	corn	inorganic	medium	750	758	growing season	dry			20.00	19	bakhsh & kanwar
drain water	ground	long	mold board	iowa USA	non-arid	corn	inorganic	medium	1041	906	annual	wet	39.04	64.65	107.19	58.06	weed & kanwar; bjorneberg et al
drain water	ground	long	mold board	iowa USA	non-arid	corn	inorganic	medium	965	906	annual	normal	18.61	34.2	62.46	63.13	weed & kanwar; bjorneberg et al
drain water	ground	long	mold board	iowa USA	non-arid	corn	inorganic	medium	744	906	annual	dry	8.08	11.95	11.72	13.26	weed & kanwar; bjorneberg et al
drain water	ground	long	mold board	iowa USA	non-arid	corn	inorganic	medium	1041	906	annual	wet	18.68	38.63	31.63	41.21	weed & kanwar; bjorneberg et al
drain water	ground	long	mold board	iowa USA	non-arid	corn	inorganic	medium	965	906	annual	normal	17.07	24.03	30.64	36.14	weed & kanwar; bjorneberg et al
drain water	ground	long	mold board	iowa USA	non-arid	corn	inorganic	medium	744	906	annual	dry	7.56	8.02	3.65	6.45	weed & kanwar; bjorneberg et al

drain water	ground	long	mold board	iowa USA	non-arid	soybean	none	medium	1041	906	annual	wet	23.04	26.08	36.47	37.95	weed & kanwar
drain water	ground	long	mold board	iowa USA	non-arid	soybean	none	medium	965	906	annual	normal	11.20	15.38	32.11	41.48	weed & kanwar
drain water	ground	long	mold board	iowa USA	non-arid	soybean	none	medium	744	906	annual	dry	6.11	7.89	3.61	7.69	weed & kanwar
leachate	ground	short	mold board	idaho USA	arid	corn	inorganic	medium	irrigation	210	annual	N/A	9.00	10			meek et al
leachate	ground	short	mold board	idaho USA	arid	wheat	inorganic	medium	irrigation	210	annual	N/A	4.00	10			meek et al
runoff	surface	long	mold board	ohio USA	non-arid	corn	inorganic	fine	562	858	annual	dry	0.70	0.3	0.08	0.01	logan et al
runoff	surface	long	mold board	ohio USA	non-arid	soybean	none	fine	562	858	annual	dry	0.40	0.6	0.03	0.02	logan et al
runoff	surface	long	mold board	ohio USA	non-arid	corn	inorganic	fine	462	858	annual	dry	0.70	0.5	0.28	0.19	logan et al
runoff	surface	long	mold board	ohio USA	non-arid	soybean	none	fine	462	858	annual	dry	1.10	1	0.59	2.34	logan et al
runoff	surface	long	mold board	ohio USA	non-arid	corn	inorganic	fine	749	858	annual	dry	4.00	0.7	1.52	0.15	logan et al
runoff	surface	long	mold board	ohio USA	non-arid	soybean	none	fine	749	858	annual	dry	0.60	0.6	0.22	0.13	logan et al
runoff	surface	long	mold board	ohio USA	non-arid	corn	inorganic	fine	1126	858	annual	wet	1.10	1.2	1.17	0.86	logan et al
runoff	surface	long	mold board	ohio USA	non-arid	soybean	none	fine	1126	858	annual	wet	1.30	1.3	1.96	0.96	logan et al

drain water	ground	long	mold board	ohio USA	non-arid	corn	inorganic	fine	562	858	annual	dry	1.30	0.01	0.1	1E-06	logan et al
drain water	ground	long	mold board	ohio USA	non-arid	soybean	none	fine	562	858	annual	dry	2.10	1.7	3.14	2.82	logan et al
drain water	ground	long	mold board	ohio USA	non-arid	corn	inorganic	fine	462	858	annual	dry	4.50	4.9	2.04	2.82	logan et al
drain water	ground	long	mold board	ohio USA	non-arid	soybean	none	fine	462	858	annual	dry	4.90	5.5	2.04	3	logan et al
drain water	ground	long	mold board	ohio USA	non-arid	corn	inorganic	fine	749	858	annual	dry	6.70	11.8	3.81	28.6	logan et al
drain water	ground	long	mold board	ohio USA	non-arid	soybean	none	fine	749	858	annual	dry	19.60	10	34.8	14.3	logan et al
drain water	ground	long	mold board	ohio USA	non-arid	corn	inorganic	fine	1126	858	annual	wet	15.00	17.8	54.8	83.4	logan et al
drain water	ground	long	mold board	ohio USA	non-arid	soybean	none	fine	1126	858	annual	wet	14.70	17.1	26.3	86.4	logan et al
runoff	surface	med	mold board	kentucky USA	non-arid	corn	inorganic	medium	rainl simulator	90 min	66 mm/h rainfall intensity	N/A	15.10	9.5	0.1	1.2	seta et al
runoff	surface	med	mold board	kentucky USA	non-arid	corn	inorganic	medium	rainl simulator	90 min	66 mm/h rainfall intensity	N/A	15.10	8	0.1	0.5	seta et al
runoff	surface	med	mold board	kentucky USA	non-arid	corn	inorganic	medium	rainl simulator	90 min	66 mm/h rainfall intensity	N/A	12.10	6.5	0.4	1.3	seta et al
runoff	surface	med	mold board	kentucky USA	non-arid	corn	inorganic	medium	rainl simulator	90 min	66 mm/h rainfall intensity	N/A	12.10	5.8	0.4	0.9	seta et al

drain water	ground	short	mold board	kentville Canada	non-arid	corn	organ ic	coars e	1224	1118	annual	normal	10.65	11.2	45.55	37.4	Fuller et al
drain water	ground	short	chisel & disk-ing	kentville Canada	non-arid	soybe an	none	coars e	996	1118	annual	dry	10.74	10.465	31.6	20.1	Fuller et al
drain water	ground	short	chisel & disk-ing	kentville Canada	non-arid	wheat	inorg anic	coars e	1109	1118	annual	normal	9.85	10.88	23.65	21.8	Fuller et al
drain water	ground	short	chisel & disk-ing	kentville Canada	non-arid	corn	organ ic	coars e	1235	1118	annual	wet	7.58	6.09	26.7	20.2	Fuller et al
drain water	ground	short	chisel & disk-ing	kentville Canada	non-arid	n/a	none	coars e	886	1118	annual	dry	7.74	6.785	23.4	20.7	Fuller et al
runoff	surface	short	mold board	kentucky USA	non-arid	corn	inorg anic	medi um	1264	1147	annual	wet			0.05	0.056	blevins et al
runoff	surface	short	mold board	kentucky USA	non-arid	corn	inorg anic	medi um	927	1147	annual	dry			1.42	1.267	blevins et al
runoff	surface	short	mold board	kentucky USA	non-arid	corn	inorg anic	medi um	909	1147	annual	dry			0.28	0.613	blevins et al
runoff	surface	short	mold board	kentucky USA	non-arid	corn	inorg anic	medi um	902	1147	annual	dry			0.18	0.468	blevins et al
runoff	surface	short	mold board	kentucky USA	non-arid	corn	inorg anic	medi um	1264	1147	annual	wet			0.05	0.032	blevins et al

runoff	surface	short	mold board	kentucky USA	non-arid	corn	inorganic	medium		927	1147	annual	dry			1.42	0.964	blevins et al
runoff	surface	short	mold board	kentucky USA	non-arid	corn	inorganic	medium		909	1147	annual	dry			0.28	0.229	blevins et al
runoff	surface	short	mold board	kentucky USA	non-arid	corn	inorganic	medium		902	1147	annual	dry			0.18	0.26	blevins et al
leaching	ground	short	mold board	maryland USA	non-arid	wheat	inorganic	medium		542	637	growing season	dry	15.55	6.95	3.50	1.5	meisinger et al
drainage	ground	short	mold board	maryland USA	non-arid	wheat	inorganic	medium		542	637	growing season	dry			2.80	2.4	meisinger et al
leachate	ground	short	mold board	maryland USA	non-arid	wheat	inorganic	medium		570	637	growing season	dry	7.35	6.25	2.20	1.3	meisinger et al
drainage	ground	short	mold board	maryland USA	non-arid	wheat	inorganic	medium		570	637	growing season	dry			1.90	1.45	meisinger et al
leachate	ground	short	mold board	maryland USA	non-arid	wheat	inorganic	medium		740	637	growing season	wet	2.85	2.95	1.40	1.3	meisinger et al
drainage	ground	short	mold board	maryland USA	non-arid	wheat	inorganic	medium		740	637	growing season	wet			3.90	3.75	meisinger et al
leachate	ground	short	mold board	new zealand	non-arid	wheat	inorganic	medium	irrigation		174	winter	N/A	42	60			fraser et al
leachate	ground	short	mold board	new zealand	non-arid	wheat	inorganic	medium	irrigation		174	winter	N/A	46.00	37			fraser et al
leachate	ground	short	mold board	new zealand	non-arid	wheat	inorganic	medium	irrigation		174	winter	N/A	42	52			fraser et al
leachate	ground	short	mold board	new zealand	non-arid	wheat	inorganic	medium	irrigation		174	winter	N/A	46.00	17			fraser et al

leachate	ground	med	mold board	michigan	non-arid	corn	inorganic	medium	855.98	805.9	annual	normal			10	12	gerakis et al
leachate	ground	med	mold board	michigan	non-arid	corn	inorganic	medium	1046.7	805.9	annual	wet			5	8	gerakis et al
leachate	ground	med	mold board	michigan	non-arid	corn	inorganic	medium	684.02	805.9	annual	dry			60	70	gerakis et al
drain water	ground	short	mold board	street ridge, canada	non-arid	corn	organic	coarse	669.9	701.3	growing season	normal	2.83	4.34	7.1	9.33	mkhabela et al
runoff	surface	short	mold board	street ridge, canada	non-arid	corn	organic	coarse	669.9	701.3	growing season	normal	2.5	3.1	17.05	10.91	mkhabela et al
drain water	ground	short	mold board	street ridge, canada	non-arid	corn	organic	coarse	712.1	701.3	growing season	normal	1.02	2.61	2.15	5.13	mkhabela et al
runoff	surface	short	mold board	street ridge, canada	non-arid	corn	organic	coarse	712.1	701.3	growing season	normal	4.65	3.91	4.61	7.17	mkhabela et al
drain water	ground	short	mold board	BEEC, canada	non-arid	soybean	organic	n/a	616.7	663.4	growing season	normal	5.17	7.03	14.57	11.72	mkhabela et al
drain water	ground	short	mold board	BEEC, canada	non-arid	soybean	organic	n/a	881.9	663.4	growing season	wet	6.91	10.54	22.37	18.19	mkhabela et al
leachate	ground	long	mold board	iowa USA	non-arid	soybean	n/a	medium	856	615	growing season	wet	1216	902			singer et al
leachate	ground	long	mold board	iowa USA	non-arid	soybean	n/a	medium	380	615	growing season	dry	1911	1240			singer et al
leachate	ground	long	mold board	iowa USA	non-arid	soybean	n/a	medium	543	615	growing season	dry	401	831			singer et al

leachate	ground	long	mold board	iowa USA	non-arid	soybean	n/a	medium	559	615	growing season	normal	294	385			singer et al
runoff	surface	med	mold board	michigan USA	non-arid	corn	inorganic	medium	963	863	annual	wet	3.5	6	19.5	21	rasse & smucker
runoff	surface	med	mold board	michigan USA	non-arid	corn	inorganic	medium	845	863	annual	normal	2	4	5	8	rasse & smucker
ground water	ground	short	mold board	delaware USA	non-arid	corn	inorganic	coarse	276 + irrigation 318, total = 594			N/A	12.70	16.7			ritter et al
ground water	ground	short	mold board	delaware USA	non-arid	corn	inorganic	coarse	330 + irrigation 272, total = 602			N/A	13.65	24.2			ritter et al
ground water	ground	short	mold board	delaware USA	non-arid	corn	inorganic	coarse	276 + irrigation 318, total = 594			N/A	12.45	15.52			ritter et al
ground water	ground	short	mold board	delaware USA	non-arid	corn	inorganic	coarse	330 + irrigation 272, total = 602			N/A	16.80	19.5			ritter et al
ground water	ground	short	mold board	delaware USA	non-arid	corn	inorganic	coarse	276 + irrigation 318, total = 594			N/A			29.1	31.6	ritter et al
ground water	ground	short	mold board	delaware USA	non-arid	corn	inorganic	coarse	330 + irrigation 272, total = 602			N/A			31.6	30.4	ritter et al
runoff	surface	short	mold board	texas USA	arid	corn	inorganic	fine	storm events			N/A			2.6	5.5	richardson & king
runoff	surface	short	mold board	texas USA	arid	wheat	inorganic	fine	storm events			N/A			4.4	13.3	richardson & king
runoff	surface	short	mold board	china	arid	wheat	inorganic	medium	storm events			N/A	1.5	1.125	0.4	0.53	wang et al
runoff	surface	short	mold board	china	arid	wheat	inorganic	medium	storm events			N/A	1.725	1.465	0.8	1.13	wang et al

drain water	ground	short	mold board	ontario Canada	non-arid	corn	inorg anic	fine	987	864	annual	wet	18.7	20.3	27.2	28.8	patni et al
drain water	ground	short	mold board	ontario Canada	non-arid	corn	inorg anic	fine	728	864	annual	dry	12.8	16.8	15.8	10.2	patni et al
drain water	ground	short	mold board	ontario Canada	non-arid	corn	inorg anic	fine	986	864	annual	wet	25.4	30.2	40.6	39.1	patni et al
drain water	ground	short	mold board	ontario Canada	non-arid	corn	inorg anic	fine	800	864	annual	normal	20.7	26.3	38.2	30	patni et al
drain water	ground	short	mold board	minnesota USA	non-arid	corn	inorg anic	medi um	3	618	growing season	dry	6	4.8	2.9	1.4	randall & iragavarapu
drain water	ground	short	mold board	minnesota USA	non-arid	corn	inorg anic	medi um	127	618	growing season	dry	9	8.1	49.4	41.1	randall & iragavarapu
drain water	ground	short	mold board	minnesota USA	non-arid	corn	inorg anic	medi um	103	618	growing season	dry	15	10.6	36.8	39.3	randall & iragavarapu
drain water	ground	short	mold board	minnesota USA	non-arid	corn	inorg anic	medi um	97	618	growing season	dry	12.5	12.8	20	17.2	randall & iragavarapu
drain water	ground	short	mold board	minnesota USA	non-arid	corn	inorg anic	medi um	125	618	growing season	dry	13.6	14	60	55.3	randall & iragavarapu
drain water	ground	med	mold board	minnesota USA	non-arid	corn	inorg anic	medi um	92	618	growing season	dry	7.8	9.2	3.2	3.8	randall & iragavarapu
drain water	ground	med	mold board	minnesota USA	non-arid	corn	inorg anic	medi um	67	618	growing season	dry	9.4	14.7	5.9	6.4	randall & iragavarapu
drain water	ground	med	mold board	minnesota USA	non-arid	corn	inorg anic	medi um	65	618	growing season	dry	13.4	11.5	5	2.5	randall & iragavarapu
drain water	ground	med	mold board	minnesota USA	non-arid	corn	inorg anic	medi um	124	618	growing season	dry	20.8	23.9	112.2	111.9	randall & iragavarapu

drain water	ground	med	mold board	minnesota USA	non-arid	corn	inorganic	medium	151	618	growing season	dry	15.3	24	113.1	138.7	randall & iragavarapu
drain water	ground	long	mold board	minnesota USA	non-arid	corn	inorganic	medium	114	618	growing season	dry	8.9	13.5	43.1	55	randall & iragavarapu
runoff	surface	long	mold board	nebraska, USA	arid	soybean	organic	medium	rain simulator			n/a			0.4	0.78	gilley et al
runoff	surface	long	mold board	nebraska, USA	arid	soybean	organic	medium	rain simulator			n/a			0.38	1.1	gilley et al
runoff	surface	long	mold board	nebraska, USA	arid	soybean	none	medium	rain simulator			n/a	1.4	0.27			gilley et al
runoff	surface	long	mold board	nebraska, USA	arid	soybean	organic	medium	rain simulator			n/a	2.43	0.71			gilley et al
runoff	surface	long	mold board	nebraska, USA	arid	soybean	organic	medium	rain simulator			n/a	2.2	0.55			gilley et al
runoff	surface	long	mold board	nebraska, USA	arid	soybean	organic	medium	rain simulator			n/a	3.63	0.84			gilley et al
runoff	surface	long	mold board	nebraska, USA	arid	soybean	organic	medium	rain simulator			n/a	1.81	0.64			gilley et al
runoff	surface	long	mold board	arkansas USA	non-arid	n/a	inorganic	N/A	n/a			n/a	0.575	0.435			sharpley et al
runoff	ground	long	mold board	arkansas USA	non-arid	n/a	inorganic	N/A	n/a			n/a	0.1	0.05			sharpley et al
runoff	ground	short	mold board	Oklahoma, USA	arid	wheat	inorganic	medium	n/a	740	annual	n/a	4.8	4			Sharpley and Smith
ground water	ground	short	mold board	Oklahoma, USA	arid	wheat	inorganic	medium	n/a	740	annual	n/a	5.1	3.2			Sharpley and Smith

ground water	ground	short	mold board	Oklahoma, USA	arid	wheat	inorganic	medium	n/a	740	annual	n/a	11.5	2			Sharpley and Smith
ground water	ground	short	mold board	Oklahoma, USA	arid	wheat	inorganic	medium	n/a	740	annual	n/a	12	1			Sharpley and Smith
ground water	ground	short	mold board	Oklahoma, USA	arid	wheat	inorganic	medium	n/a	740	annual	n/a	12	2.2			Sharpley and Smith
ground water	ground	med	mold board	Oklahoma, USA	arid	wheat	inorganic	medium	n/a	740	annual	n/a	19	1.5			Sharpley and Smith
ground water	ground	med	mold board	Oklahoma, USA	arid	wheat	inorganic	medium	n/a	740	annual	n/a	16	1.5			Sharpley and Smith
ground water	ground	med	mold board	Oklahoma, USA	arid	wheat	inorganic	medium	n/a	740	annual	n/a	20	2			Sharpley and Smith
ground water	ground	short	mold board	Oklahoma, USA	arid	wheat	inorganic	medium	n/a			n/a	0.5	5			Sharpley and Smith
ground water	ground	short	mold board	Oklahoma, USA	arid	wheat	inorganic	medium	n/a			n/a	1.5	4.3			Sharpley and Smith
ground water	ground	short	mold board	Oklahoma, USA	arid	wheat	inorganic	medium	n/a			n/a	7	3			Sharpley and Smith
ground water	surface	short	mold board	Oklahoma, USA	arid	wheat	inorganic	medium	900	750	annual	wet	2.04	1.38	3.16	1.48	smith et al
ground water	surface	short	mold board	Oklahoma, USA	arid	wheat	inorganic	medium	600	730	annual	dry	1.47	1.03	0.83	0.7	smith et al
runoff	ground	med	mold board	wisconsin USA	non-arid	corn	inorganic	medium	990	812	annual	wet	4.2	9.5	14	31	brye & norman
runoff	ground	short	rototiller	china	arid	corn	inorganic	medium	irrigation			n/a	10	10	15.1	13.4	huang et al

leachate	ground	short	rototiller	china	arid	corn	organic	medium	irrigation		n/a	7.6	5	5.3	3.4	huang et al
leachate	ground	short	rototiller	china	arid	corn	organic	medium	irrigation		n/a	20	20	26.5	24	huang et al
leachate	ground	short	rototiller	china	arid	corn	inorganic	medium	irrigation		n/a	37.5	35	51.5	39	huang et al
leachate	ground	short	rototiller	china	arid	corn	organic	medium	irrigation		n/a	25	20	32.1	20.9	huang et al
leachate	ground	short	rototiller	china	arid	corn	organic	medium	irrigation		n/a	66	64	76.6	52.9	huang et al
leachate	ground	short	rototiller	china	arid	corn	inorganic	medium	irrigation		n/a	50	37.5	11.8	6.3	huang et al
leachate	ground	short	rototiller	china	arid	corn	organic	medium	irrigation		n/a	22	23	2.3	1.6	huang et al
leachate	ground	short	rototiller	china	arid	corn	organic	medium	irrigation		n/a	70	70	16.5	12.2	huang et al
leachate	ground	short	rototiller	china	arid	corn	inorganic	medium	irrigation		n/a	34	25	27.9	18.8	huang et al
leachate	ground	short	rototiller	china	arid	corn	organic	medium	irrigation		n/a	10	5	7.3	3.2	huang et al
leachate	ground	short	rototiller	china	arid	corn	organic	medium	irrigation		n/a	75	55	61.7	39.6	huang et al
leachate	ground	short	rototiller	china	arid	corn	inorganic	medium	irrigation		n/a	37.5	30	29.8	19	huang et al
leachate	ground	short	rototiller	china	arid	corn	organic	medium	irrigation		n/a	12.5	10	12.4	7	huang et al

leachate	ground	short	rototiller	china	arid	corn	organic	medium	irrigation		n/a	60	50	46.3	28.5	huang et al
leachate	ground	short	rototiller	china	arid	wheat	inorganic	medium	irrigation		n/a	10	10	3.9	3.2	huang et al
leachate	ground	short	rototiller	china	arid	wheat	organic	medium	irrigation		n/a	7.6	5	2.7	1.7	huang et al
leachate	ground	short	rototiller	china	arid	wheat	organic	medium	irrigation		n/a	20	20	12.5	8.4	huang et al
leachate	ground	short	rototiller	china	arid	wheat	inorganic	medium	irrigation		n/a	37.5	35	3.9	3.1	huang et al
leachate	ground	short	rototiller	china	arid	wheat	organic	medium	irrigation		n/a	25	20	1.8	1.3	huang et al
leachate	ground	short	rototiller	china	arid	wheat	organic	medium	irrigation		n/a	66	64	16.8	13.3	huang et al
leachate	ground	short	rototiller	china	arid	wheat	inorganic	medium	irrigation		n/a	50	37.5	31.5	25.3	huang et al
leachate	ground	short	rototiller	china	arid	wheat	organic	medium	irrigation		n/a	22	23	18.8	12.8	huang et al
leachate	ground	short	rototiller	china	arid	wheat	organic	medium	irrigation		n/a	70	70	60.7	47.2	huang et al
leachate	ground	short	rototiller	china	arid	wheat	inorganic	medium	irrigation		n/a	34	25	5.1	2.2	huang et al
leachate	ground	short	rototiller	china	arid	wheat	organic	medium	irrigation		n/a	10	5	1.7	0.5	huang et al
leachate	ground	short	rototiller	china	arid	wheat	organic	medium	irrigation		n/a	75	55	17.2	8.6	huang et al

leachate	ground	short	rototiller	china	arid	wheat	inorganic	medium	irrigation	n/a	37.5	30	27.6	13.7	huang et al
leachate	ground	short	rototiller	china	arid	wheat	organic	medium	irrigation	n/a	12.5	10	10.4	8.1	huang et al
leachate	ground	short	rototiller	china	arid	wheat	organic	medium	irrigation	n/a	60	50	47.3	34.7	huang et al
leachate	ground	short	chisel	wisconsin USA	non-arid	corn	inorganic	medium	450	dry	23	28.9	5	10	brye et al
leachate	ground	short	chisel	wisconsin USA	non-arid	corn	inorganic	medium	433	dry	11.25	5.25	40	32	brye et al
leachate	ground	short	chisel	wisconsin USA	non-arid	corn	inorganic	medium	480.1	normal	8.07	4.85	5	10	brye et al
leachate	surface	med	Disk-ing	nebraska USA	arid	corn	organic	medium	rain simulator at 64 mm/hour for 1 hour	n/a	26.6	22.4			eghball & gilley
leachate	surface	med	Disk-ing	nebraska USA	arid	corn	organic	medium	rain simulator at 64 mm/hour for 1 hour	n/a	47.2	26.9			eghball & gilley
runoff	surface	med	Disk-ing	nebraska USA	arid	corn	inorganic	medium	rain simulator at 64 mm/hour for 1 hour	n/a	24.9	28.6			eghball & gilley
runoff	surface	med	Disk-ing	nebraska USA	arid	corn	none	medium	rain simulator at 64 mm/hour for 1 hour	n/a	22.8	24.7			eghball & gilley
runoff	surface	med	Disk-ing	nebraska USA	arid	corn	organic	medium	rain simulator at 64 mm/hour for 2 hour	n/a	22.6	23.3			eghball & gilley
runoff	surface	med	Disk-ing	nebraska USA	arid	corn	organic	medium	rain simulator at 64 mm/hour for 2 hour	n/a	26.5	20.7			eghball & gilley
runoff	surface	med	Disk-ing	nebraska USA	arid	corn	inorganic	medium	rain simulator at 64 mm/hour for 2 hour	n/a	35.8	25			eghball & gilley

runoff	surface	med	Disk-ing	nebraska USA	arid	corn	none	medium	rain simulator at 64 mm/hour for 2 hour			n/a	23.7	23.3			eghball & gilley
runoff	surface	med	Disk-ing	nebraska USA	arid	corn	n/a	medium	rain simulator at 64 mm/hour for 1 hour			n/a	27	46.8			eghball & gilley
runoff	surface	med	Disk-ing	nebraska USA	arid	corn	n/a	medium	rain simulator at 64 mm/hour for 2 hour			n/a	23.3	23.8			eghball & gilley
runoff	surface	short	Disk-ing	iowa USA	non-arid	corn	organic	fine	rain simulator at 64 mm/hour for 1 hour			n/a	27.1	26.8			eghball et al
runoff	surface	short	Disk-ing	iowa USA	non-arid	corn	organic	fine	rain simulator at 64 mm/hour for 1 hour			n/a	26	25.6			eghball et al
runoff	surface	short	Disk-ing	iowa USA	non-arid	corn	inorganic	fine	rain simulator at 64 mm/hour for 1 hour			n/a	30.6	30.2			eghball et al
runoff	surface	short	Disk-ing	iowa USA	non-arid	corn	inorganic	fine	rain simulator at 64 mm/hour for 1 hour			n/a	31.2	23.9			eghball et al
runoff	surface	short	Disk-ing	iowa USA	non-arid	corn	none	fine	rain simulator at 64 mm/hour for 1 hour			n/a	27.1	18.5			eghball et al
runoff	surface	short	Disk-ing	iowa USA	non-arid	corn	none	fine	rain simulator at 64 mm/hour for 1 hour			n/a	26	19.6			eghball et al
runoff	surface	short	Disk-ing	iowa USA	non-arid	corn	n/a	fine	rain simulator at 64 mm/hour for 1 hour			n/a			0.052	0.604	eghball et al
runoff	surface	short	Disk-ing	iowa USA	non-arid	corn	n/a	fine	rain simulator at 64 mm/hour for 1 hour			n/a			2.641	4.447	eghball et al
runoff	ground	short	Disk-ing	ames Iowa USA	non-arid	corn	inorganic	medium	766	813	growing season	normal	12.5	9			al-kaisi & litch
runoff	ground	short	chisel	ames Iowa USA	non-arid	corn	inorganic	medium	766	813	growing season	normal	12.5	15			al-kaisi & litch

leachate	ground	short	chisel	ames Iowa USA	non-arid	soybean	none	medium	713	813	growing season	dry	7.5	12.5		al-kaisi & litch
leachate	ground	short	chisel	ames Iowa USA	non-arid	soybean	none	medium	713	813	growing season	dry	7.5	10.625		al-kaisi & litch
leachate	ground	short	chisel	nashua Iowa USA	non-arid	corn	inorganic	medium	832	864	growing season	normal	10	17.5		al-kaisi & litch
leachate	ground	short	chisel	nashua Iowa USA	non-arid	corn	inorganic	medium	832	864	growing season	normal	10	11		al-kaisi & litch
leachate	ground	short	chisel	nashua Iowa USA	non-arid	soybean	none	medium	711	864	growing season	dry	15	17		al-kaisi & litch
leachate	ground	short	chisel	nashua Iowa USA	non-arid	soybean	none	medium	711	864	growing season	dry	15	20		al-kaisi & litch
leachate	ground	short	chisel & disk-ing	kentucky USA	non-arid	corn	organic	medium	1016	1148	annual	dry	6.77	6.63		stoddard et al
leachate	ground	short	chisel & disk-ing	kentucky USA	non-arid	corn	organic	medium	1269	1148	annual	wet	6.425	5.207		stoddard et al
leachate	ground	short	chisel & disk-ing	kentucky USA	non-arid	corn	organic	medium	1150	1148	annual	normal	11.125	9.325		stoddard et al
leachate	ground	short	chisel & disk-ing	kentucky USA	non-arid	corn	organic	medium	1029	1148	annual	dry	18.4	14.5		stoddard et al

leachate	ground	short	chisel & disk-ing	pensylvania USA	non-arid	corn	inorganic	medium	n/a			n/a	3.8	1.7	23	11	zhu et al
leachate	ground	short	chisel & disk-ing	pensylvania USA	non-arid	corn	inorganic	medium	n/a			n/a	9	7.4	49	45	zhu et al
leachate	ground	short	chisel & disk-ing	pensylvania USA	non-arid	corn	inorganic	medium	n/a			n/a	22.2	24.7	121	140	zhu et al
leachate	ground	short	chisel & disk-ing	pensylvania USA	non-arid	soybean	none	medium	n/a			n/a	5.3	6.8	18	23	zhu et al
leachate	ground	short	chisel & disk-ing	pensylvania USA	non-arid	soybean	none	medium	n/a			n/a	9.1	7.4	30	31	zhu et al
leachate	ground	short	chisel & disk-ing	pensylvania USA	non-arid	soybean	none	medium	n/a			n/a	22.7	29.8	34	96	zhu et al
leachate	ground	long	Disk-ing	nebraska USA	non-arid	wheat	organic	medium	rain simulator			n/a	8.08	11.49			gilley et al
leachate	ground	med	Disk-ing	nebraska USA	non-arid	wheat	organic	medium	rain simulator			n/a	11.33	16			gilley et al
runoff	surface	long	chisel	iowa USA	non-arid	corn	inorganic	medium	1030	840	annual	wet	9.4	11.4	25	32	bakhsh et al

runoff	surface	long	chisel	iowa USA	non-arid	corn	inorganic	medium	750	840	annual	dry	8.1	8.8	5	9	bakhsh et al
drain water	ground	long	chisel	iowa USA	non-arid	corn	inorganic	medium	800	840	annual	normal	10.9	13.5	10	12	bakhsh et al
drain water	ground	long	chisel	iowa USA	non-arid	corn	inorganic	medium	680	840	annual	dry	15.3	13.9	8	8	bakhsh et al
drain water	ground	long	chisel	iowa USA	non-arid	corn	inorganic	medium	750	840	annual	dry	12.6	10.5	7	7	bakhsh et al
drain water	ground	long	chisel	iowa USA	non-arid	corn	inorganic	medium	980	840	annual	wet	11.8	12.1	24	34	bakhsh et al
drain water	ground	long	chisel	iowa USA	non-arid	corn	inorganic	medium	1030	840	annual	wet	9.3	9.3	46	33	bakhsh et al
drain water	ground	long	chisel	iowa USA	non-arid	corn	inorganic	medium	750	840	annual	dry	6.3	9.3	10	3	bakhsh et al
drain water	ground	long	chisel	iowa USA	non-arid	corn	inorganic	medium	800	840	annual	normal	12.7	15.5	25	10	bakhsh et al
drain water	ground	long	chisel	iowa USA	non-arid	corn	inorganic	medium	680	840	annual	dry	12.8	13	14	6	bakhsh et al
drain water	ground	long	chisel	iowa USA	non-arid	corn	inorganic	medium	750	840	annual	dry	12.3	12.4	17	6	bakhsh et al
drain water	ground	long	chisel	iowa USA	non-arid	corn	inorganic	medium	980	840	annual	wet	10.9	12.7	40	24	bakhsh et al
drain water	ground	long	chisel	iowa USA	non-arid	soybean	none	medium	1030	840	annual	wet	5.9	6.3	23	30	bakhsh et al
drain water	ground	long	chisel	iowa USA	non-arid	soybean	none	medium	750	840	annual	dry	4.6	6.7	3	5	bakhsh et al

drain water	ground	long	chisel	iowa USA	non-arid	soybean	none	medium	800	840	annual	normal	8.5	10.3	9	18	bakhsh et al
drain water	ground	long	chisel	iowa USA	non-arid	soybean	none	medium	680	840	annual	dry	15.7	12.9	8	6	bakhsh et al
drain water	ground	long	chisel	iowa USA	non-arid	soybean	none	medium	750	840	annual	dry	7.9	7.6	6	11	bakhsh et al
drain water	ground	long	chisel	iowa USA	non-arid	soybean	none	medium	980	840	annual	wet	11.8	11.1	23	23	bakhsh et al
drain water	ground	long	chisel	iowa USA	non-arid	soybean	none	medium	1030	840	annual	wet	6.5	11.5	37	23	bakhsh et al
drain water	ground	long	chisel	iowa USA	non-arid	soybean	none	medium	750	840	annual	dry	4.8	6.2	6	3	bakhsh et al
drain water	ground	long	chisel	iowa USA	non-arid	soybean	none	medium	800	840	annual	normal	9	10.9	23	10	bakhsh et al
drain water	ground	long	chisel	iowa USA	non-arid	soybean	none	medium	680	840	annual	dry	12.4	15.1	13	6	bakhsh et al
drain water	ground	long	chisel	iowa USA	non-arid	soybean	none	medium	750	840	annual	dry	7.3	6.8	16	4	bakhsh et al
drain water	ground	long	chisel	iowa USA	non-arid	soybean	none	medium	980	840	annual	wet	9.7	11.9	26	24	bakhsh et al
drain water	ground	long	chisel	iowa USA	non-arid	corn	inorganic	medium	1141	758	growing season	wet	39.00	54	96	89	kanwar et al; baksh & kanwar; singh & kanwar
drain water	ground	long	chisel	iowa USA	non-arid	corn	inorganic	medium	892	758	growing season	wet	19.00	28	56	68	kanwar et al; baksh & kanwar; singh & kanwar

drain water	ground	long	chisel	iowa USA	non-arid	corn	inorganic	medium	741	758	growing season	normal	11.00	15	18	17	kanwar et al; baksh & kanwar; singh & kanwar
drain water	ground	long	chisel	iowa USA	non-arid	corn	inorganic	medium	1141	758	growing season	wet	23.00	28	33	47	kanwar et al; baksh & kanwar; singh & kanwar
drain water	ground	long	chisel	iowa USA	non-arid	corn	inorganic	medium	892	758	growing season	wet	17.00	21	27	33	kanwar et al; baksh & kanwar; singh & kanwar
drain water	ground	long	chisel	iowa USA	non-arid	corn	inorganic	medium	741	758	growing season	normal	8.00	10	4	15	kanwar et al; baksh & kanwar; singh & kanwar
drain water	ground	long	chisel	iowa USA	non-arid	soybean	none	medium	1141	758	growing season	wet	22.00	33	33	46	kanwar et al; baksh & kanwar; singh & kanwar
drain water	ground	long	chisel	iowa USA	non-arid	soybean	none	medium	892	758	growing season	wet	11.00	15	29	41	kanwar et al; baksh & kanwar; singh & kanwar
drain water	ground	long	chisel	iowa USA	non-arid	soybean	none	medium	741	758	growing season	normal	9.00	12	4	7	kanwar et al; baksh & kanwar; singh & kanwar
drain water	ground	long	chisel	iowa USA	non-arid	corn	inorganic	medium	1141	758	growing season	wet	39.00	44	96	75	kanwar et al; baksh & kanwar; singh & kanwar
drain water	ground	long	chisel	iowa USA	non-arid	corn	inorganic	medium	892	758	growing season	wet	19.00	21	56	56	kanwar et al; baksh & kanwar; singh & kanwar

drain water	ground	long	chisel	iowa USA	non-arid	corn	inorganic	medium	741	758	growing season	normal	11.00	11	18	10	kanwar et al; baksh & kanwar; singh & kanwar
drain water	ground	long	chisel	iowa USA	non-arid	corn	inorganic	medium	1141	758	growing season	wet	23.00	22	33	27	kanwar et al; baksh & kanwar; singh & kanwar
drain water	ground	long	chisel	iowa USA	non-arid	corn	inorganic	medium	892	758	growing season	wet	17.00	18	27	26	kanwar et al; baksh & kanwar; singh & kanwar
drain water	ground	long	chisel	iowa USA	non-arid	corn	inorganic	medium	741	758	growing season	normal	8.00	10	4	11	kanwar et al; baksh & kanwar; singh & kanwar
drain water	ground	long	chisel	iowa USA	non-arid	soybean	none	medium	1141	758	growing season	wet	22.00	24	33	30	kanwar et al; baksh & kanwar; singh & kanwar
drain water	ground	long	chisel	iowa USA	non-arid	soybean	none	medium	892	758	growing season	wet	11.00	12	29	29	kanwar et al; baksh & kanwar; singh & kanwar
drain water	ground	long	chisel	iowa USA	non-arid	soybean	none	medium	741	758	growing season	normal	9.00	11	4	9	kanwar et al; baksh & kanwar; singh & kanwar
drain water	ground	long	chisel	iowa USA	non-arid	corn	inorganic	medium	1030	840	annual	wet	9.30	9.3	45.8	32.8	bakhsh & kanwar
drain water	ground	long	chisel	iowa USA	non-arid	corn	inorganic	medium	750	840	annual	dry	8.20	9.3	13.5	2.7	bakhsh & kanwar
drain water	ground	long	chisel	iowa USA	non-arid	corn	inorganic	medium	800	840	annual	normal	12.70	15.5	25.2	10.5	bakhsh & kanwar

drain water	ground	long	chisel	iowa USA	non-arid	corn	inorganic	medium	680	840	annual	dry	12.80	13	13.7	6.3	bakhsh & kanwar
drain water	ground	long	chisel	iowa USA	non-arid	corn	inorganic	medium	750	840	annual	dry	12.30	12.4	16.6	6.3	bakhsh & kanwar
drain water	ground	long	chisel	iowa USA	non-arid	corn	inorganic	medium	980	840	annual	wet	10.90	12.7	39.7	23.6	bakhsh & kanwar
drain water	ground	long	chisel	iowa USA	non-arid	soybean	none	medium	1030	840	annual	wet	6.50	11.5	37.1	32.3	bakhsh & kanwar
drain water	ground	long	chisel	iowa USA	non-arid	soybean	none	medium	750	840	annual	dry	4.80	6.2	5.6	3.4	bakhsh & kanwar
drain water	ground	long	chisel	iowa USA	non-arid	soybean	none	medium	800	840	annual	normal	9.00	10.9	23.1	10.2	bakhsh & kanwar
drain water	ground	long	chisel	iowa USA	non-arid	soybean	none	medium	680	840	annual	dry	12.40	15.1	12.9	5.7	bakhsh & kanwar
drain water	ground	long	chisel	iowa USA	non-arid	soybean	none	medium	750	840	annual	dry	7.30	6.8	15.7	3.7	bakhsh & kanwar
drain water	ground	long	chisel	iowa USA	non-arid	soybean	none	medium	980	840	annual	wet	9.70	11.9	25.9	24.5	bakhsh & kanwar
drain water	ground	long	chisel	iowa USA	non-arid	corn	inorganic	medium	1041	906	annual	wet	39.04	54.61	107.19	99.92	weed & kanwar; bjorneberg et al
drain water	ground	long	chisel	iowa USA	non-arid	corn	inorganic	medium	965	906	annual	normal	18.61	27.98	62.46	76.06	weed & kanwar; bjorneberg et al
drain water	ground	long	chisel	iowa USA	non-arid	corn	inorganic	medium	744	906	annual	dry	8.08	10.49	11.72	13.43	weed & kanwar; bjorneberg et al
drain water	ground	long	chisel	iowa USA	non-arid	corn	inorganic	medium	1041	906	annual	wet	18.68	32.67	31.63	51.16	weed & kanwar; bjorneberg et al

drain water	ground	long	chisel	iowa USA	non-arid	corn	inorganic	medium	965	906	annual	normal	17.07	20.59	30.64	36.49	weed & kanwar; bjorneberg et al
drain water	ground	long	chisel	iowa USA	non-arid	corn	inorganic	medium	744	906	annual	dry	7.56	27.08	3.65	4.86	weed & kanwar; bjorneberg et al
drain water	ground	long	chisel	iowa USA	non-arid	soybean	none	medium	1041	906	annual	wet	23.04	14.49	36.47	52.36	weed & kanwar; bjorneberg et al
drain water	ground	long	chisel	iowa USA	non-arid	soybean	none	medium	965	906	annual	normal	11.20	7.08	32.11	46.15	weed & kanwar; bjorneberg et al
drain water	ground	long	chisel	iowa USA	non-arid	soybean	none	medium	744	906	annual	dry	6.11	6.99	3.61	11.71	weed & kanwar; bjorneberg et al
drain water	ground	long	chisel	iowa USA	non-arid	corn	inorganic	medium	1041	906	annual	wet	39.04	83.41	107.19	43.61	weed & kanwar; bjorneberg et al
drain water	ground	long	chisel	iowa USA	non-arid	corn	inorganic	medium	965	906	annual	normal	18.61	67.59	62.46	20.72	weed & kanwar; bjorneberg et al
drain water	ground	long	chisel	iowa USA	non-arid	corn	inorganic	medium	744	906	annual	dry	8.08	0.89	11.72	0.86	weed & kanwar; bjorneberg et al
drain water	ground	long	chisel	iowa USA	non-arid	corn	inorganic	medium	1041	906	annual	wet	18.68	33.94	31.63	24.5	weed & kanwar; bjorneberg et al
drain water	ground	long	chisel	iowa USA	non-arid	corn	inorganic	medium	965	906	annual	normal	17.07	29.82	30.64	18.67	weed & kanwar; bjorneberg et al
drain water	ground	long	chisel	iowa USA	non-arid	corn	inorganic	medium	744	906	annual	dry	7.56	2.84	3.65	3.21	weed & kanwar; bjorneberg et al
drain water	ground	long	chisel	iowa USA	non-arid	soybean	none	medium	1041	906	annual	wet	23.04	30.34	36.47	21.26	weed & kanwar; bjorneberg et al
drain water	ground	long	chisel	iowa USA	non-arid	soybean	none	medium	965	906	annual	normal	11.20	32.21	32.11	11.68	weed & kanwar; bjorneberg et al

drain water	ground	long	chisel	iowa USA	non-arid	soybean	none	medium	744	906	annual	dry	6.11	4.49	3.61	3.43	weed & kanwar; bjorneberg et al
runoff	surface	short	chisel	texas USA	arid	n/a	inorganic	fine	average 3 years			n/a			2.5	5.5	chicester & richardson
runoff	surface	short	chisel	texas USA	arid	n/a	inorganic	fine	average 3 years			n/a			1.9	1.6	chicester & richardson
runoff	surface	short	chisel	texas USA	arid	n/a	inorganic	fine	average 3 years			n/a			5.9	10.4	chicester & richardson
runoff	surface	short	chisel	virginia USA	non-arid	n/a	inorganic	medium	rainl simulator			n/a	2.72	0.61			mostaghimi et al
runoff	surface	short	chisel	virginia USA	non-arid	n/a	inorganic	medium	rainl simulator			n/a	3.72	1.16			mostaghimi et al
runoff	surface	short	chisel	virginia USA	non-arid	n/a	inorganic	medium	rainl simulator			n/a	4.14	0.62			mostaghimi et al
runoff	surface	short	chisel	virginia USA	non-arid	n/a	inorganic	medium	rainl simulator			n/a	1.07	1.23			mostaghimi et al
runoff	surface	short	chisel	virginia USA	non-arid	n/a	inorganic	medium	rainl simulator			n/a	1.2	2.62			mostaghimi et al
runoff	surface	short	chisel	virginia USA	non-arid	n/a	inorganic	medium	rainl simulator			n/a	1.06	1.52			mostaghimi et al
runoff	surface	short	chisel	virginia USA	non-arid	n/a	inorganic	medium	rainl simulator			n/a	2	4.51			mostaghimi et al
runoff	surface	short	chisel	virginia USA	non-arid	n/a	inorganic	medium	rainl simulator			n/a	3.41	5.28			mostaghimi et al
runoff	surface	short	chisel	virginia USA	non-arid	n/a	inorganic	medium	rainl simulator			n/a	2.38	2.57			mostaghimi et al

runoff	surface	short	chisel	kansas USA	arid	soybean	none	fine	901	970	annual	normal	0.7	0.9	1.3	1.1	zeimen et al
runoff	surface	med	chisel	kansas USA	arid	soybean	none	fine	848	970	annual	dry	1.1	2.8	0.5	1	zeimen et al
runoff	surface	short	chisel	kansas USA	arid	soybean	none	fine	935	970	annual	normal	0.3	0.4	1	0.9	zeimen et al
runoff	surface	short	chisel	kansas USA	arid	soybean	none	fine	1096	970	annual	wet	1.3	1.6	1	1	zeimen et al
runoff	surface	short	chisel	kansas USA	arid	soybean	none	fine	935	970	annual	normal	0.5	0.2	2.5	0.7	zeimen et al
runoff	surface	short	chisel	kansas USA	arid	soybean	none	fine	1096	970	annual	wet	1.1	1	1.5	2.2	zeimen et al
runoff	surface	short	paraplow	ohio USA	non-arid	corn	inorganic	medium	828	965	annual	dry	17.4	5	7.5	1.2	owens & edwards
runoff	surface	short	paraplow	ohio USA	non-arid	corn	inorganic	medium	940	965	annual	normal	15.45	15.65	11.05	9.15	owens & edwards
runoff	surface	short	paraplow	ohio USA	non-arid	corn	inorganic	medium	945	965	annual	normal	5	9.3	1.55	2.3	owens & edwards
runoff	surface	short	paraplow	ohio USA	non-arid	soybean	none	medium	987	965	annual	normal	2.1	1.55	1.25	1	owens & edwards
runoff	surface	short	paraplow	ohio USA	non-arid	soybean	none	medium	941	965	annual	normal	0.55	0.65	0.4	0.25	owens & edwards
runoff	surface	med	paraplow	ohio USA	non-arid	soybean	none	medium	950	965	annual	normal	1.05	2.8	1.25	2.5	owens & edwards
runoff	surface	short	paraplow	ohio USA	non-arid	corn	inorganic	medium	828	965	annual	dry	17.4	14.25	7.5	4.4	owens & edwards

runoff	surface	short	paraplow	ohio USA	non-arid	corn	inorganic	medium	940	965	annual	normal	15.45	28.1	11.05	25.55	owens & edwards
runoff	surface	short	paraplow	ohio USA	non-arid	corn	inorganic	medium	945	965	annual	normal	5	35.3	1.55	4.95	owens & edwards
runoff	surface	short	paraplow	ohio USA	non-arid	soybean	none	medium	987	965	annual	normal	2.1	4.9	1.25	4.7	owens & edwards
runoff	surface	short	paraplow	ohio USA	non-arid	soybean	none	medium	941	965	annual	normal	0.55	2.3	0.4	2.15	owens & edwards
runoff	surface	med	paraplow	ohio USA	non-arid	soybean	none	medium	950	965	annual	normal	1.05	2.95	1.25	3.85	owens & edwards
runoff	surface	med	chisel	minnesota USA	non-arid	corn	inorganic	medium	414 & 780			n/a	56	81			joshi et al
runoff	surface	med	chisel	minnesota USA	non-arid	corn	organic	medium	414 & 780			n/a	43	57			joshi et al
leachate	ground	med	chisel	minnesota USA	non-arid	corn	organic	medium	414 & 780			n/a	14	8			joshi et al
leachate	ground	med	chisel	minnesota USA	non-arid	corn	organic	medium	414 & 780			n/a	8	15			joshi et al
leachate	ground	med	chisel	minnesota USA	non-arid	corn	n/a	medium	414	581	growing season	dry	28.4	64.5			joshi et al
leachate	ground	med	chisel	minnesota USA	non-arid	corn	n/a	medium	780	581	growing season	wet	57.9	75.6			joshi et al
leachate	ground	short	Disk-ing & harrowing	nigeria	non-arid	corn	inorganic	coarse	1217	1357	april-oct	dry	3.35	1.83			lal et al

leachate	ground	short	Disk-ing & harrowing	nigeria	non-arid	corn	inorganic	coarse	1890	1357	april-oct	wet	1.84	1.77	lal et al
runoff	surface	short	Disk-ing & harrowing	nigeria	non-arid	corn	inorganic	coarse	1100	1357	april-oct	dry	2.86	2.48	lal et al
runoff	surface	short	Disk-ing & harrowing	nigeria	non-arid	corn	inorganic	coarse	1500	1357	april-oct	wet	3.78	1.69	lal et al
runoff	surface	short	Disk-ing & harrowing	nigeria	non-arid	corn	inorganic	coarse	1385	1357	april-oct	normal	1.3	0.74	lal et al
runoff	surface	short	Disk-ing & harrowing	nigeria	non-arid	corn	inorganic	coarse	1217	1357	april-oct	dry	1.72	1.79	lal et al
runoff	surface	short	Disk-ing & harrowing	nigeria	non-arid	corn	inorganic	coarse	1890	1357	april-oct	wet	3.23	1.91	lal et al
runoff	surface	short	Disk-ing & harrowing	nigeria	non-arid	corn	inorganic	coarse	1100	1357	april-oct	dry	2.2	1.72	lal et al
runoff	surface	short	Disk-ing &	nigeria	non-arid	corn	inorganic	coarse	1500	1357	april-oct	wet	5.79	1.94	lal et al

			harrowing													
runoff	surface	short	Disk-ing & harrowing	nigeria	non-arid	corn	inorganic	coarse	1385	1357	april-oct	normal	2.26	1.17		lal et al
runoff	surface	short	Disk-ing & harrowing	nigeria	non-arid	corn	inorganic	coarse	1217	1357	april-oct	dry	2.61	1.43		lal et al
runoff	surface	short	Disk-ing & harrowing	nigeria	non-arid	corn	inorganic	coarse	1890	1357	april-oct	wet	2.12	2.04		lal et al
runoff	surface	short	Disk-ing & harrowing	nigeria	non-arid	corn	inorganic	coarse	1100	1357	april-oct	dry	2.17	1.93		lal et al
runoff	surface	short	Disk-ing & harrowing	nigeria	non-arid	corn	inorganic	coarse	1500	1357	april-oct	wet	1.26	2.12		lal et al
runoff	surface	short	Disk-ing & harrowing	nigeria	non-arid	corn	inorganic	coarse	1385	1357	april-oct	normal	1.09	0.81		lal et al
runoff	surface	short	Disk-ing & harrowing	nigeria	non-arid	corn	inorganic	coarse	1217	1357	april-oct	dry	2.41	1.94		lal et al

runoff	surface	short	Disk-ing & harrowing	nigeria	non-arid	corn	inorganic	coarse	1890	1357	april-oct	wet	2.08	1.08		lal et al
runoff	surface	short	Disk-ing & harrowing	nigeria	non-arid	corn	inorganic	coarse	1100	1357	april-oct	dry	2.69	1.87		lal et al
runoff	surface	short	Disk-ing & harrowing	nigeria	non-arid	corn	inorganic	coarse	1500	1357	april-oct	wet	1.36	1.37		lal et al
runoff	surface	short	Disk-ing & harrowing	nigeria	non-arid	corn	inorganic	coarse	1385	1357	april-oct	normal	0.92	1		lal et al
runoff	surface	short	Disk-ing & harrowing	nigeria	non-arid	corn	inorganic	coarse	1217	1357	april-oct	dry	2.48	1.87		lal et al
runoff	surface	short	Disk-ing & harrowing	nigeria	non-arid	corn	inorganic	coarse	1890	1357	april-oct	wet	1.94	2.25		lal et al
runoff	surface	short	Disk-ing & harrowing	nigeria	non-arid	corn	inorganic	coarse	1100	1357	april-oct	dry	2.77	3.6		lal et al
runoff	surface	short	Disk-ing &	nigeria	non-arid	corn	inorganic	coarse	1500	1357	april-oct	wet	2.43	1.62		lal et al

			harrowing													
runoff	surface	short	Disk-ing & harrowing	nigeria	non-arid	corn	inorganic	coarse	1385	1357	april-oct	normal	0.75	0.64		lal et al
runoff	surface	short	Disk-ing & harrowing	nigeria	non-arid	corn	inorganic	coarse	1217	1357	april-oct	dry	1.71	1.41		lal et al
runoff	surface	short	Disk-ing & harrowing	nigeria	non-arid	corn	inorganic	coarse	1890	1357	april-oct	wet	2.03	1.64		lal et al
runoff	surface	short	Disk-ing & harrowing	nigeria	non-arid	corn	inorganic	coarse	1100	1357	april-oct	dry	1.54	1.79		lal et al
runoff	surface	short	Disk-ing & harrowing	nigeria	non-arid	corn	inorganic	coarse	1500	1357	april-oct	wet	1.47	1.47		lal et al
runoff	surface	short	Disk-ing & harrowing	nigeria	non-arid	corn	inorganic	coarse	1385	1357	april-oct	normal	1.21	0.78		lal et al

Supplementary Table S3. Calculation from Lajeunesse et al. (2015)

	Leaching concentration (NT)	Runoff concentration (NT)	Leaching concentration (CT)	Runoff concentration (CT)	Leaching load (NT)	Runoff load (NT)	Leaching load (CT)	Runoff load (CT)
Overall	3.3554	7.8109	4.3752	7.3838	14.8939	5.1686	13.9851	5.1240
Dryland	7.9584	4.7375	6.9478	4.3698	6.1349	4.7877	5.9063	3.2637
Non-dryland	9.9089	6.9073	14.9312	6.5125	13.6626	4.2558	13.1217	4.1229
Fine-texture	5.2177	4.3224	4.9033	4.9260	3.8265	4.9315	3.4651	3.2555
Medium-texture	16.2959	10.8680	15.7066	10.7055	13.3590	4.069	12.601	3.7432
Coarse-texture	5.2732	12.0465	6.1438	13.5507	7.8461	1.7408	8.3248	4.8334
Dry year	5.1547	3.6666	8.9040	4.4737	5.7357	3.1980	4.7717	3.6915
Normal year	14.6570	3.8510	8.7038	3.0337	8.1166	3.6301	7.9079	3.6770
Wet year	11.8913	7.4063	10.0960	6.8638	11.7409	3.0227	14.1006	2.6239
Corn	14.6344	7.8448	13.9262	7.1899	11.9455	4.1995	11.4617	3.8541
Soybean	14.0854	7.6098	12.4351	5.4871	12.1825	6.3758	8.1807	5.3787
Wheat	6.9547	2.3982	5.7786	1.9263	4.4604	2.5894	4.2436	1.4756
Short duration	10.2574	5.3996	10.5340	5.1060	8.4454	4.1585	7.1300	3.7839
Medium duration	8.1510	7.0773	6.3635	6.3766	5.0581	2.4277	4.3341	2.6628
Long duration	15.9081	3.8030	12.0155	1.9139	11.2084	3.4592	11.6498	3.5524
Organic fertilizer	6.3817	3.9645	6.6270	4.3370	7.5498	1.3635	7.8148	1.9680
No fertilizer	7.2176	2.6266	6.3699	2.9311	6.5340	6.1615	5.2331	4.6215
Inorganic fertilizer	18.0824	6.2754	15.5303	5.6247	12.2449	4.6689	11.5314	4.0724
